# FQStat: a parallel architecture for very high-speed assessment of sequencing quality metrics

**DOI:** 10.1186/s12859-019-3015-y

**Published:** 2019-08-15

**Authors:** Sree K. Chanumolu, Mustafa Albahrani, Hasan H. Otu

**Affiliations:** Department of Electrical and Computer Engineering, University of Nebraska-Lincoln, Lincoln, NE 68588 USA

**Keywords:** Sequence quality, FASTQ, Parallel programming

## Abstract

**Background:**

High throughput DNA/RNA sequencing has revolutionized biological and clinical research. Sequencing is widely used, and generates very large amounts of data, mainly due to reduced cost and advanced technologies. Quickly assessing the quality of giga-to-tera base levels of sequencing data has become a routine but important task. Identification and elimination of low-quality sequence data is crucial for reliability of downstream analysis results. There is a need for a high-speed tool that uses optimized parallel programming for batch processing and simply gauges the quality of sequencing data from multiple datasets independent of any other processing steps.

**Results:**

FQStat is a stand-alone, platform-independent software tool that assesses the quality of FASTQ files using parallel programming. Based on the machine architecture and input data, FQStat automatically determines the number of cores and the amount of memory to be allocated per file for optimum performance. Our results indicate that in a core-limited case, core assignment overhead exceeds the benefit of additional cores. In a core-unlimited case, there is a saturation point reached in performance by increasingly assigning additional cores per file. We also show that memory allocation per file has a lower priority in performance when compared to the allocation of cores. FQStat’s output is summarized in HTML web page, tab-delimited text file, and high-resolution image formats. FQStat calculates and plots read count, read length, quality score, and high-quality base statistics. FQStat identifies and marks low-quality sequencing data to suggest removal from downstream analysis. We applied FQStat on real sequencing data to optimize performance and to demonstrate its capabilities. We also compared FQStat’s performance to similar quality control (QC) tools that utilize parallel programming and attained improvements in run time.

**Conclusions:**

FQStat is a user-friendly tool with a graphical interface that employs a parallel programming architecture and automatically optimizes its performance to generate quality control statistics for sequencing data. Unlike existing tools, these statistics are calculated for multiple datasets and separately at the “lane,” “sample,” and “experiment” level to identify subsets of the samples with low quality, thereby preventing the loss of complete samples when reliable data can still be obtained.

**Electronic supplementary material:**

The online version of this article (10.1186/s12859-019-3015-y) contains supplementary material, which is available to authorized users.

## Background

High throughput DNA/RNA sequencing has revolutionized our understanding of and approach to biological and clinical research. Advancements in its technology and reduced cost have made sequencing available to a broad research and industry base [1]. Be it RNAseq, ChIPseq, de novo genome sequencing, resequencing, or metagenomics, sequencing data in the range of giga-to-tera base throughput per experiment is typically generated by an average-sized research lab or company. The National Center for Biotechnology Information’s (NCBI) Sequence Read Archive (SRA) database [2], one of the major publicly available molecular sequencing databases, has increased in size from about 20 G bases (Gb) to 20 peta bases (Pb) in approximately ten years. We have seen sequencing efforts across the board in the evolutionary tree, starting with viruses and bacteria and continuing all the way to humans resulting in thousands of datasets [3, 4]. Hence, there is a constant need across a wide spectrum of biotechnology stakeholders for improved data storage and analysis methods that are able to handle large datasets in a reasonable amount of time.

From the early Sanger-based sequencing methods to the present next-generation sequencing (NGS) and beyond, sequencing has targeted the reconstruction of whole genomes and understanding the dynamics of molecules in a variety of biological samples, including specific model organisms, nonmodel organisms [5], and clinical samples [6, 7]. With the availability of huge sequencing repositories, higher-level studies have emerged, such as meta-analysis (large-scale, integrated analysis on multiple datasets) [8], multiparameter combinatorial approaches, both in terms of the environment and the organism, and translational medicine efforts [9]. On the other hand, the decrease in sequencing cost has far outpaced Moore’s Law [10], rendering significant challenges to data storage and computational analysis. Therefore, whether it is an experiment generating new sequencing data or a study that attempts to analyze existing sequencing datasets, it is not atypical to be required to analyze hundreds or thousands of sequencing data samples. As a result, approaches making use of high computing clusters, such as parallel computing, have been an important aspect of dealing with increased sequencing data analysis and storage needs [11, 12].

One of the critical steps of sequencing data analysis workflows is the quality control (QC). Eliminating low-quality data samples based on QC analysis vastly improves the accuracy and reliability of the downstream data analysis results [13]. Furthermore, identifying data quality problems and carefully inspecting them provides feedback on the experimental procedures to pinpoint the aspects of the protocol that have led to quality problems. Projects that involve new datasets or reanalysis of existing datasets and large research and sequencing centers need to quickly assess the QC of hundreds or thousands of samples. Several sequencing QC tools have been developed using either serial [14–21] or parallel [22–28] architecture. However, the QC tools with parallel architecture do not optimize system performance by dynamic core or thread assignment; they just assign one core per file if multiple cores are available or divide a file into multiple cores and process the files sequentially. These approaches do not optimize memory assignment per file, nor do they process multiple files in parallel where each file is analyzed by multiple processors. We have improved on this facet in FQStat by probing system architecture and automatically optimizing the core/memory assignment per file in a dynamic fashion.

Most of the existing QC tools provide more capabilities than just QC assessment, such as variant reporting [14, 23], adapter detection [15], fusion transcript detection [16], integration of different NGS data [17], trimming [27], or read alignment [24, 28]. Although these approaches are helpful, they force the users to commit to analysis steps other than QC assessment to calculate simple QC parameters. In order to assess the QC of the sequencing data, the user needs to use the tool’s sequence processing steps and obtain the QC statistics for the processed data obtained using the tool. Furthermore, these programs lack the ability to take more than one dataset (e.g., raw and trimmed) as input and compare their respective QC parameters. This feature, for example, comes in handy when the quality of two different experiments is compared or when data trimming and filtering demonstrate improvement. FQStat can take two datasets as input and focus only on a comparative QC assessment of the two datasets, independent from any other sequence processing steps.

In a typical sequencing project, a sample is run on multiple lanes; and the reads coming from different lanes are combined [29]. The reason for this is to still be able to obtain reliable data even if one or more lanes have failed. None of the aforementioned QC tools provide lane-level statistics to let the user pick and choose among the lanes that have performed satisfactorily. This becomes critical when sample-level statistics might warrant discarding the complete sequencing data for the sample, whereas lane-level statistics may identify lanes with reliable data that may be combined to represent the sample. Furthermore, in some analysis settings, experiment-level, not sample-level QC assessment is required. Most of the QC tools cited provide sample-level analysis and do not provide a “big picture,” experiment-level assessment; and those tools that do provide experiment-level assessment, do not provide sample-level analysis results. Finally, samples that have low QC parameters are not flagged in the existing QC tools to warn the user of potential samples to be discarded. Therefore, there is a need for a quick QC assessment tool that is not tied to other sequence processing steps, simply assesses the QC parameters for one or more datasets, automatically optimizes parallel architecture, provides lane and experiment level statistics, and flags low-quality lanes and/or samples. We believe FQStat fills this gap. A feature comparison with similar QC tools is provided in Table 1.

**Table 1:** Feature comparison between FQStat and similar QC tools

Feature\Tool	FQStat	FaQCs	NGS QC Box	NGS QC ToolKit	RNA-QC-Chain	ClinQC
Lane-level statistics	Yes	No	No	No	No	No
Experiment-level statistics	Yes	No	Yes	No	No	No
Trimming and filtering	No	Yes	Yes	Yes	Yes	Yes
Comparison of multiple datasets	Yes	No	No	No	No	No
Sample flagging	Yes	No	No	No	No	No
Parallel programing	Yes	Yes	Yes	Yes	Yes	Yes
Support compressed files	Yes	Yes	Yes	Yes	No	No
Graphical output	Yes	Yes	No	Yes	Yes	Yes
Multithreading	Yes	Yes	No	Yes	Yes	No
Multiple processing for a single file (MPSF)	Yes	Yes/No*	No	Yes	Yes	No
Multiple processing for multiple files (MPMF)	Yes	No	Yes	Yes	No	Yes
Concurrent implementation of MPSF and MPMF	Yes	No	No	No	No	No
Memory requirement	No	Yes	No	No	No	No
Memory optimization	Yes	No	N/A	N/A	Yes	Yes
Automated resource optimization	Yes	No	No	No	Yes	No

*FaQCs divides a single file into split files and each split file is run on different processors but if the number of processors is more than the number of split files, then a split file is not processed by multiple processes

### Implementation

FQStat is coded in Python 3.7.3® (http://www.python.org) using the multiprocessing package and has a graphical user interface (GUI) where input/output files and program parameters can be configured. In order to avoid any mix-up from the outputs of different processes, FQStat uses lock protection on threads, the outputs of which are then assembled to generate FQStat’s final results. FQStat can also be executed using the command-line, enabling its use as part of scripts that handle batch processing. FQStat works with both single-end and paired-end FASTQ sequencing data, processes samples run on multiple lanes, and accepts one or two datasets (e.g., raw and trimmed) as input that involve the same samples. This information is conveyed to FQStat by arranging the sequencing files in a certain folder structure and by following a specific file naming convention as explained in the online tutorial. In consideration of the ever-increasing sequencing file sizes and numbers, we have implemented FQStat to handle both regular and compressed files. FQStat calculates and plots read count, read length, quality score, and high-quality base statistics. These statistics are calculated at the lane, sample, and experiment (dataset) levels; and the percent difference between the two datasets is noted. Experiment-level statistics are calculated in two ways: (i) lane resolution, where lane-level data are assumed to form the experiment; and (ii) sample resolution, where sample-level data are assumed to form the experiment. A high-quality base is defined as a base with a quality score above Q, which is a parameter that can be adjusted by the user. The default value for Q is 25.

The results of FQStat are stored in an HTML file. The tables and graphs shown in the HTML file are also saved as tab-delimited text files and high-resolution images, respectively. Image resolution can be adjusted by the user. FQStat identifies and marks low-quality sequencing data to suggest removal from downstream analysis. For each calculated statistic, lane- or sample-level sequencing data with an absolute z-score above 1.5 are flagged to warrant further inspection by the user. The 1.5 z-score cut-off can be changed by the user as an input parameter. In order to optimize system performance, FQStat probes its environment to obtain the number of available cores, available memory, and the number and size of input files to be analyzed. The simulations reported in this report were run on a single rack server with 768 GB of RAM and dual Intel® Xeon® E7–8870 CPU with 144 cores.

### Workflow

**Fig. 1:**
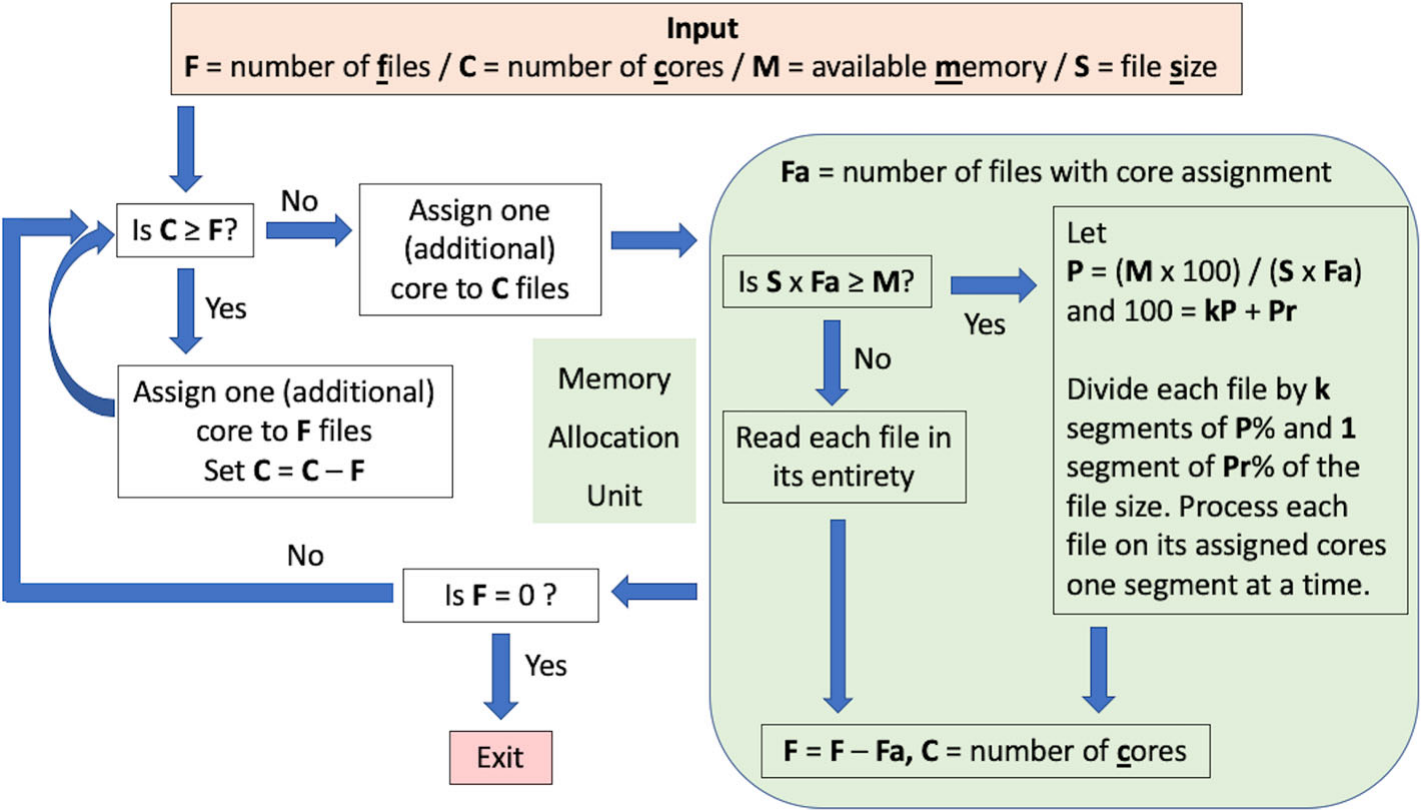
Overall workflow of FQStat. FQStat automatically optimizes the number of cores assigned to a file and the file split size (segments). System resources regarding the available cores and memory are utilized to full capacity

The overall workflow of FQStat is shown in Fig. 1. The choice of core and memory allocations described in this section is based on our experiments, which are explained in the subsequent sections of this manuscript. FQStat first compares the number of files with the number of available cores. If the former is larger, it assigns one file per core and iteratively processes the files until all of the files are analyzed. If the latter is larger, the cores are divided among the files as evenly as possible as long as the number of cores assigned to a file does not exceed *max_core*. The *max_core* is a parameter that defines the number of cores above which core assignment to a file decreases the overall run time for the file. The default value for *max_core* is 55, but this value can be adjusted by the user.

If the available memory is less than the total file size to be analyzed, each file is split into segments such that the sum of the total segment size for each file (analyzed at a given instance by all the cores) equals the available memory. At each iteration, only the segment size of the file is read into the memory prior to processing. This way, FQStat is free from any memory requirements and can handle large file sizes and large numbers of files with limited memory resources. Here, we present three examples of core/memory assignment where **F** is the total number of files, **C** is the total number of available cores, **S** is the file size, and **M** is the available memory. Although file size may change from file to file and FQStat calculates the size of individual files for automatic performance optimization, we assume each FASTQ file has the same size, **S**, for the purpose of these examples.

*Example Case 1*, **F** = 30, **C** = 100, **S** = 2GB, **M** = 128GB: 20 files are assigned to 3 cores and 10 files are assigned to 4 cores. Each file is read (processed) in its entirety.

*Example Case 2*, **F** = 250, **C** = 40, **S** = 2GB, **M** = 128GB: Each file is assigned to 1 core, and each file is read in its entirety. After this is repeated for 6 iterations (6 × 40 = 240), the remaining (250–240 = 10) files in the following (final) iteration are assigned to 4 cores each; and each file is read in its entirety.

*Example Case 3*, **F** = 250, **C** = 100, **S** = 2GB, **M** = 60GB: Each file is assigned to 1 core, and each file is split into 4 segments such that the first three segments are 30% and the last segment is 10% of the total file size. This way, all 60GB of the available memory are used in the first three iterations. This whole procedure is repeated 2 times (2 × 100 = 200); and in the following (last) iteration, the remaining (250–200 = 50) files are assigned to 2 cores each. In this last iteration, each file is split into two segments such that the first segment is 60% of the file size, and the second segment is 40% of the file size.

## Results

### Effect of file split size and number of cores per file

**Fig. 2:**
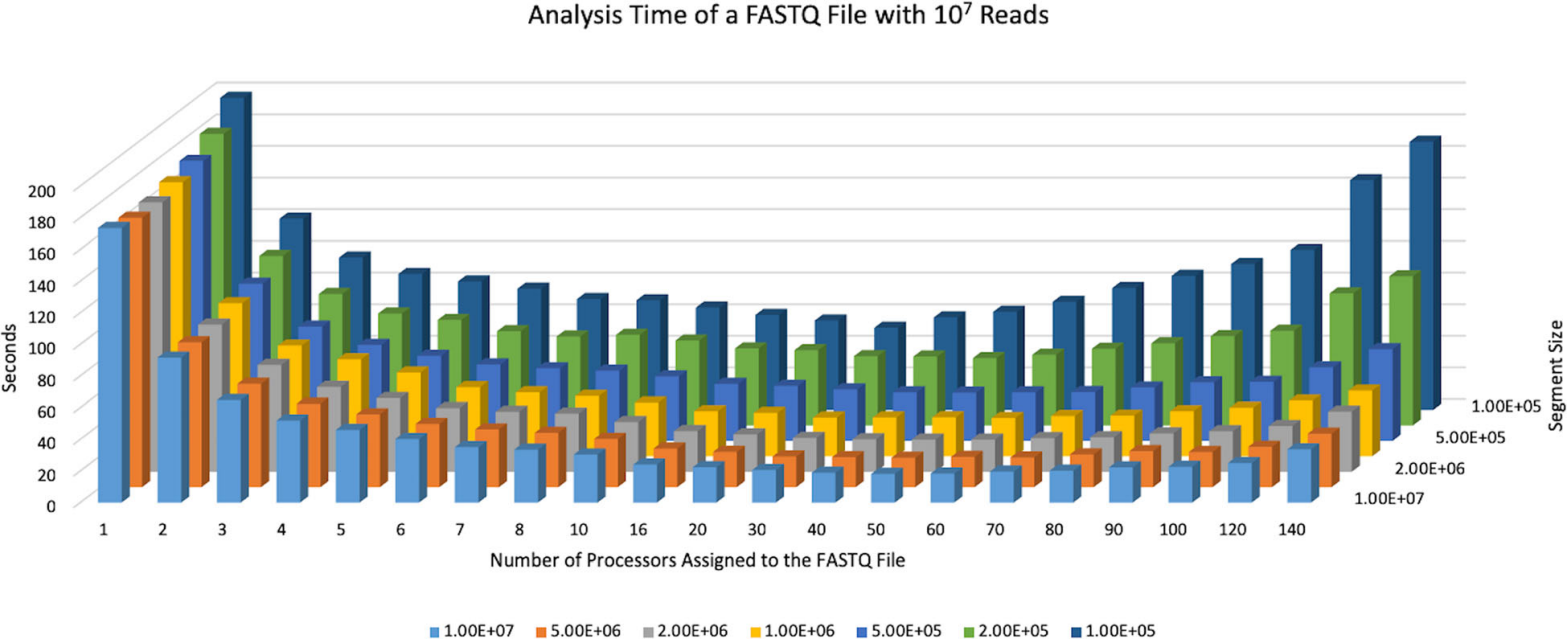
Time performance of FQStat using a test file with 10^7^ reads (~ 75 bp read length). The performance is shown as a function of file split (segment) size and the number of cores assigned to the file. FQStat performs better with increased segment size, and the improvement in performance due to increased core assignment reaches a saturation point beyond which additional core assignment deteriorates program performance

In order to determine the effect of segment size (into which a FASTQ file is split) and the number of cores assigned to a file on run time, we used a test FASTQ file with 10 million reads from a real RNA-seq experiment that has targeted a read length of 75 bp. The file is either read in its entirety (segment size = 1 × 10^7^ reads) or split into segments of 5 × 10^6^, 2 × 10^6^, 1 × 10^6^, 5 × 10^5^, 2 × 10^5^, and 1 × 10^5^ reads. That is, when the segment size is 2 × 10^6^ reads, for example, the file is split into 5 segments, each segment is processed in parallel using the C cores assigned to the file, and the 5 segments are iteratively analyzed. The results summarized in Fig. 2 show that when a file is read in its entirety (i.e., segment size = file size), irrespective of the number of cores assigned to a file, the optimum performance was achieved. This is expected as splitting a file into segments and consolidating the results provides a time overhead compared to reading a file in its entirety.

Increasing the number of cores assigned to a file, irrespective of the segment size, decreases the run time of the program until a certain point beyond which additional core assignment works against program performance. The time overhead incurred in parallel programming by using multiple cores involve task start-up and task termination times, synchronization, data communications, etc. Our results indicate that increased core assignment provides, at some point, enough time overhead that erases the performance benefit obtained by having more cores. For each segment size, the time required to analyze the test file follows a valley pattern (Fig. 2). The minimum point, that is the optimum number of cores assigned to a file, is reached between 50 and 60 cores. Therefore, we picked the *max_core* parameter in FQStat to be 55, which can be adjusted by the user. Although this limit is unlikely to be reached in real-life applications, FQStat still provides protection against performance deterioration by assigning too many cores per file. Also evident from Fig. 2 is the fact that the degree of performance deterioration by increased core assignment is more pronounced as the file is split into smaller segments. For example, when 140 cores are assigned to the test file using a 1 × 10^5^-read segment size, the run time is ~ 86% of the one-core case. On the other hand, when the file is read in its entirety, the 140-core case takes only ~ 19% as much as that of the one-core case to run.

In addition to the case shown in Fig. 2, we have run additional simulations where the file size ranged from 10^6^ to 10^8^ reads, the segment size ranged from 10^5^ to 10^8^ reads, the number of available cores ranged from 10 to 140, and each case tried for read lengths of 75 bp, 100 bp, and 150 bp. Our results show that the conclusions attained in the case for Fig. 2 remains true for the different combinations of files size, segment size, number of cores, and average read length. Despite showing slight variations among different combinations, run-times worsen after the number of cores assigned to a file exceed a threshold. These results are summarized in Additional files 1, 2, 3.

### Performance gain per assigned core

In order to see the degree of performance increase by additional core assignment, we defined the term “performance gain” (PG) using the one-core case as the baseline. The PG for the case when C cores are assigned to a file is defined as the ratio of the “time to process the file with one core” to “C times the time to process the file with C cores.” For example, if C = 2 and we use exactly half of the time it takes to analyze the file with one core, then PG = 1. However, if the time to analyze the file with two cores is less than half of the time it takes to analyze the file with one core, then PG > 1, otherwise PG < 1. When PG > 1, it implies improved gains in performance with respect to the one-core case; and the larger the PG, the better the improvement in performance.

**Fig. 3:**
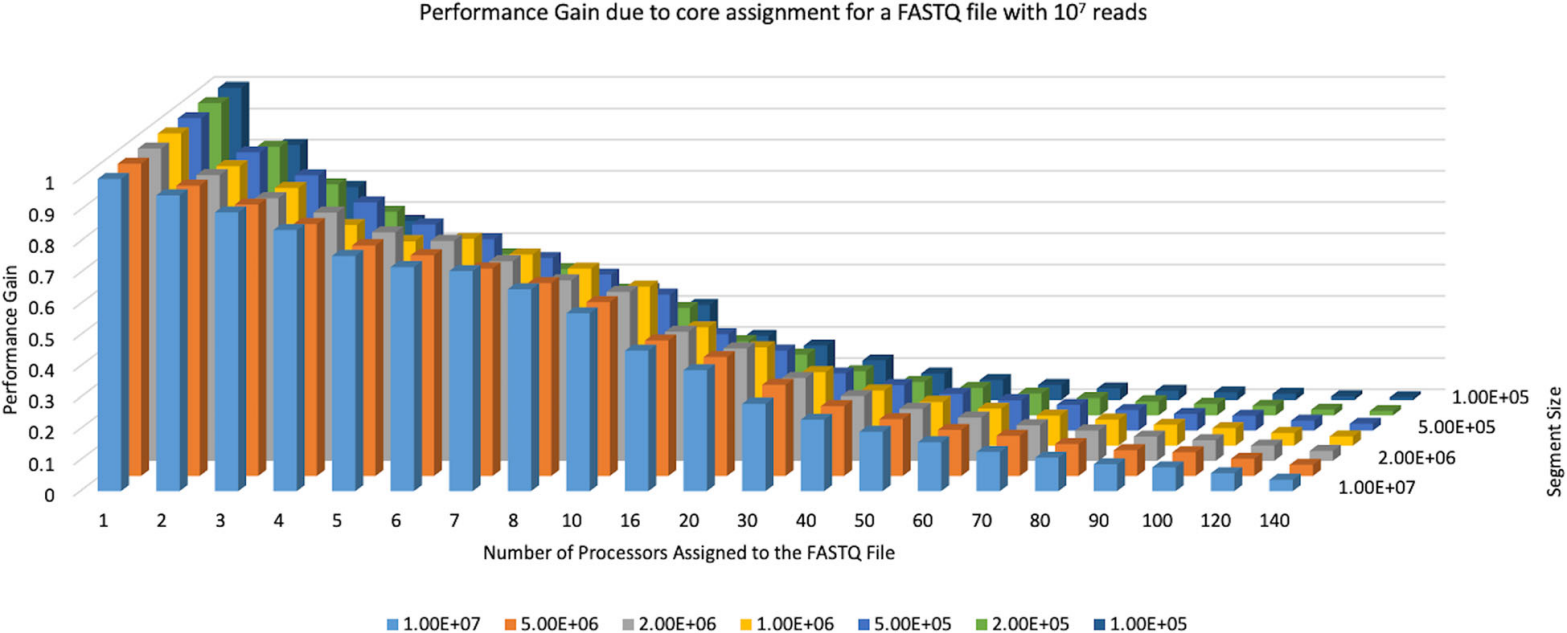
Performance Gain (PG) with increased core assignment. The PG for the test file (10^7^ reads, ~ 75 bp read length) as a function of the number of assigned cores and segment size. A PG less than 1 implies that the run time using C cores is more than 1/C of the one-core run time. Additional core assignments do not achieve at par PGs and loss in PG becomes worse with decreased segment size

We used the same test file (10^7^ reads with ~ 75 bp per read) and segment sizes, as described in the previous subsection, to calculate the PG for varying core assignments. The results, summarized in Fig. 3, show that the performance gain due to increased core assignment is always less than 1 and inversely proportional to the number of assigned cores. This is somewhat expected as the benefit obtained by additional cores is undermined by the overhead incurred by core assignment and data consolidation. Another outcome of this exercise was the reassurance of the benefit obtained by increased segment size. For almost all core assignment cases, the highest PG is obtained when the segment size equals the file size; and PGs show a monotonically decreasing behavior with decreasing segment size, i.e., more splits of the file.

The two reported experiments imply that if the number of files exceeds the number of cores, then we should assign one core per file and process the files in groups until all files are analyzed. On the other hand, if there are more cores than files, then the cores should be divided among the files as evenly as possible. Furthermore, if there is sufficient available memory, then the files should be read in their entirety for processing (no splits). If the system memory is not large enough to read the files in full, then the segment size into which the files are split should be as large as possible, constrained by the memory limitations. For example, if we have 20 files and 5 cores, our results suggest that it does not help to analyze the files one at a time, processed in parallel across the 5 cores; but rather the files should be analyzed 5 at a time, each assigned to one core, processed in parallel.

Similar to the previous subsection, we repeated the simulation shown in Fig. 3 for the aforementioned varying levels of file size, segment size, number of cores, and read length. For each combination, our simulation results indicate that the conclusions based on Fig. 3 hold true (Additional files 4, 5, 6), i.e., PG decreases with additional core assignment.

There still remains the question, “Does the performance loss due to additional core assignment exceed the performance loss due the file splits?” We emphasize that “performance loss due to additional core assignment” does not mean longer run times with the additional core assignment (as long as they are less than *max_core*, run times are always improved with additional cores), but we mean the observed PGs that are < 1 (Fig. 2). On the other hand, we know that splitting the files deteriorates program performance as well. However, if we have to split files, is it better to assign multiple cores per file without splitting the file than to assign one core per file and split it? For example, if we have 20 files, 4 cores, 2GB per file, and 4 GB of available memory, should we still use one core per file and split the files into two segments of 50%; or should we use two cores per file and read the files in their entirety? We know that using two cores per file does not improve the run time by a factor of two, but we also know that splitting the files deteriorates program performance. We try to answer this question in the next subsection.

### File split versus core assignment

In order to determine the trade-off between a file split and a decrease in PG with increased core assignment in a core-limited and/or memory-limited case, we constructed the following experiment. We generated 100 files of 2 GB each and limited the resources available to FQStat to 40 cores and 20 GB. This way, even if we assign one core per file, we cannot read all of the files in their entirety, as that would require 80 GB of available memory. Therefore, we devised three analysis approaches where either one core per file is assigned (and the files are spilt), or the files are read in their entirety (but assigned to multiple cores), or a compromise is made between the two cases. This simulation was repeated for read lengths of 75 bp, 100 bp, and 150 bp.

The results of this experiment are shown in Table 2. When we assigned 1 core per file, it was necessary to read the files in 4 splits (i.e., each segment size was 25% of the file size). Similarly, when we assigned 2 cores per file, the files needed to be processed in segment sizes of 50%. Finally, assigning 4 cores per file let us read the files in their entirety. Our results suggest that irrespective of the read length, the overhead incurred by splitting the files is less than that of core assignment as the fastest performance was achieved when one processor per file was assigned despite splitting the files into four segments for processing (due to memory limitations). Therefore, in a core- and memory-limited case, FQStat assigns one core per file and splits the files based on memory limitations (Fig. 1).

**Table 2:** Assessing the file split versus core assignment trade-off

Time (sec)			
Strategy	1 core per file 25% segment size	2 cores per file 50% segment size	4 cores per file 100% segment size
Read Length			
75 bp	2253	2625	2922
100 bp	2262	2684	2931
150 bp	2363	2851	2986

The input to FQStat was 100 FASTQ files each with 2 GB of size. These 100 files were generated using three different read lengths: 75 bp, 100 bp, 150 bp. The available resources were kept at 40 cores and 40 GB of memory. For each read length/analysis strategy combination, the total run time is shown in seconds

Following the results of our experiments for performance optimization, we set up the FQStat processing strategy, as described in Fig. 1.

## Discussion

Given the volume of newly generated and reanalyzed sequencing data and the importance of QC analysis, FQStat uses a parallel programming architecture to introduce the following improvements: (i) automatic configuration of system parameters (e.g., core assignment and file segmentation) for optimum performance; (ii) analysis of multiple data sets for comparative assessment of QC parameters; (iii) not being coupled with other preprocessing steps (e.g., read mapping or low quality base trimming) for an easy-to-use, simple, and fast calculation of QC parameters only; (iv) generating analysis results separately at the lane-, sample-, and experiment-level so the users can pick and choose high quality subsets of the sample and/or experiment data; (v) flagging low quality lanes and/or samples that warrant further analysis; (vi) generating publication quality output figures and tables. FQStat handles both paired-end and single-end sequencing data run on single or multiple lanes. Such input data options and parameters are described by the user either using the GUI or the command-line version of FQStat. The former version is intended for a single experiment analysis providing the user a friendly interface whereas the latter is intended to be included in more complex workflows or batch processing. The details of the installation and usage of FQStat along with step-by-step screenshots of each process are included in the online tutorial.

### Sample output

We tried FQStat on real RNA-seq data available at NCBI’s SRA database with BioProject ID: PRJNA492713. The RNA-seq portion of this data set was comprised of 8 samples where each sample was run on 4 lanes using the Illumina NextSeq 500® platform. The target read length in the data set was 75 bp and the experiment was performed using paired-end sequencing. We processed the raw data using FASTQC (v. 0.11.5) [30] and Trimmomatic (v 0.38) [31]. This way, we obtained two datasets to be used in FQStat: “raw” (the dataset as downloaded from SRA) and “trimmed” (FASTQ files processed using FASTQC and Trimmomatic). We applied FASTQC separately for each lane of a sample and identified overrepresented sequences and other adapter and similar technical sequences, which were subsequently removed by Trimmomatic (v 0.38) in the palindrome mode, based on default alignment detection and scoring parameters. Maximum information quality filtering followed by a minimum average read quality threshold of 25 was used in Trimmomatic for low quality base filtering. Following technical sequence and low-quality base removal, reads that were shorter than 40 bp were filtered out.

**Fig. 4:**
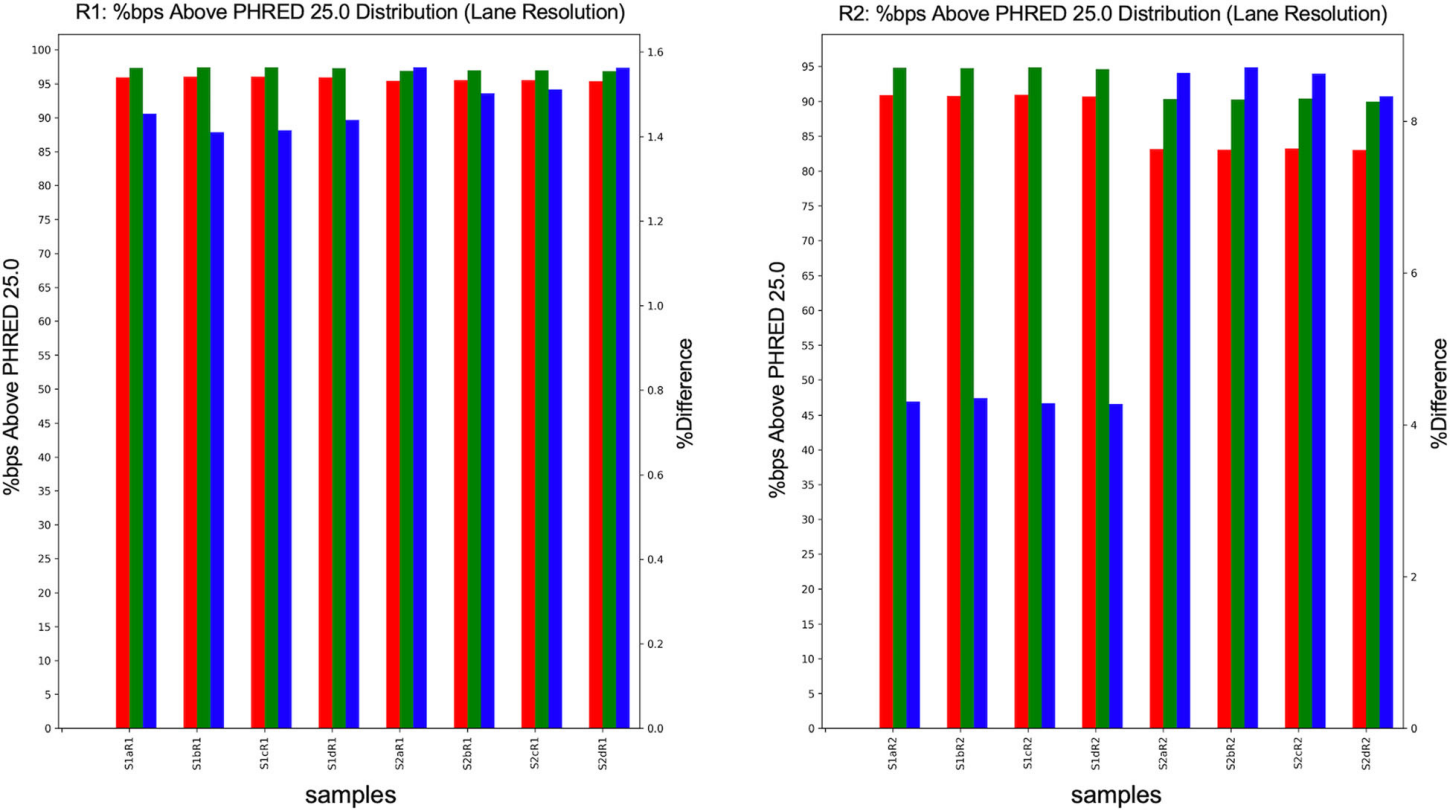
Sample FQStat output displaying high-quality bp %. The percentage of bps that exceeds the default high-quality bp score of 25 are displayed for “raw” and “trimmed” FASTQ files. The percent difference between the two datasets is shown in the secondary y-axis. The results are shown for two samples (S1 and S2), for both forward and reverse reads (R1 and R2), for all four lanes (a, b, c, and d)

On the FQStat web page (http://otulab.unl.edu/FQStat) we provide the full results of FQStat analysis on 2 samples from the described RNA-seq dataset. In Fig. 4, we provide a sample plot of FQStat for demonstration purposes. We chose to us the “high-quality bp %” feature of FQStat as this feature is rarely observed in existing QC tools and nicely demonstrates the improvements attained by the trimming and filtering step. We used default parameters in FQStat, and hence reported the percentage of bps that exceeded the quality score of 25 in both “raw” and “trimmed” FASTQ files. The results indicate that our trimming and filtering strategy has improved the high-quality bp percentage by about 1.5% on average for both samples on the forward reads (R1). However, for the reverse reads (R2), sample 1 (S1) has shown about 4.3% increase in high quality bp percentage, whereas this increase jumps up to about 8.6% for sample 2 (S2) R2 reads, following trimming and filtering. The starting high quality bp percentage (raw) for both samples in R2 is low (~ 90% in S1 and 83% in S2), which probably results in higher % increases due to trimming; and warrants a more careful look at the R2 data, providing feedback to the experimental phase of the project.

### Comparison with other QC tools

We assessed FQStat’s performance in comparison with the five QC tools listed in Table 1 using the aforementioned test RNA-seq dataset that consisted of 100 2GB fastq files. In order to have a large dataset size, we multiplexed these files to be used as their paired-end counterparts, as well as the trimmed and filtered versions, resulting in 400 files. The test files used in this manuscript can be found on the FQStat website (http://otulab.unl.edu/FQStat). All of the tools used in our simulations implemented a parallel programming architecture and provided basic QC parameters. The test was done using two configurations. The first configuration was 140 cores with 768 GB of available memory aimed at mimicking a medium-to-high resource. The second configuration represented a more typical, low-to-medium resource and had 16 cores with 128 GB of available memory. As shown in Table 3, FQStat outperforms most other QC tools that utilize a parallel architecture.

**Table 3:** Run time for QC tools with parallel architecture using the test RNA-seq dataset

Time (sec)						
Resource\Tool	FQStat	FaQCs	NGS QC Box	NGS QC ToolKit	RNA-QC-Chain	ClinQC
140 cores, 768 GB memory	1811	13,519	1560	13,051	8273	39,270
16 cores, 128 GB memory	6272	18,200	3668	62,371	15,980	94,882

Where applicable, we used only the quality control aspect of the programs, omitting other processing steps (e.g., trimming and filtering) that would increase run time. NGS QC Box was the only program that outperformed our program, FQStat. However, NGS QC Box (run in “quick mode” in our simulations) did not generate QC statistics at the sample level but rather generated these statistics at the experiment level. In other words, while FQStat generated detailed statistics for each of the 400 files, NGS QC Box generated 4 values for each statistic, one for each “experiment”: Raw Pair1, Raw Pair2, Trimmed Pair1, and Trimmed Pair2. As generating an ensemble statistic for the entire 400 files requires dramatically less time than generating individual statistics for each of the 400 files, we believe FQStat’s effective performance is better than that of NGS QC Box. We also note that compared to NGS QC Box, FQStat attains a higher ratio of reduction in run-time when the system resources are improved (~ 3.5 vs. ~ 2.4), which may be attributable FQStat’s ability to optimize system resources.

## Conclusions

Despite the popularity of DNA/RNA sequencing, there is still need for a high-speed tool that uses parallel programming to gauge the quality of the data without committing to other sequence processing steps, automatically optimizes system performance, processes more than one dataset comparatively, and analyzes the data at lane-, sample-, and experiment-levels. We developed FQStat to address these issues and provide this platform-independent tool with a graphical user interface that is easy to use. FQStat works faster than similar QC tools and identifies and marks low-quality data to be further inspected by the user. The output can be easily monitored through two HTML files (one for graphs and one for tables), and the results are also stored in tab-delimited text files and publication-ready figure formats. We believe FQStat can be used by any sequencing pipeline to assist with experimental and analysis workflows, and we plan to continuously improve FQStat by incorporating additional QC parameters in its analysis core.

## Additional files

Additional file 1: Effect of file split size and number of cores per file. Read Length = 75 bp (PDF 1417 kb)

Additional file 2: Effect of file split size and number of cores per file. Read Length = 100 bp (PDF 1424 kb)

Additional file 3: Effect of file split size and number of cores per file. Read Length = 150 bp (PDF 1462 kb)

Additional file 4: Performance gain per assigned core. Read Length = 75 bp (PDF 1079 kb)

Additional file 5: Performance gain per assigned core. Read Length = 100 bp (PDF 1082 kb)

Additional file 6: Performance gain per assigned core. Read Length = 150 bp (PDF 1083 kb)

## Data Availability

The software and data used in this publication are available at: http://otulab.unl.edu/FQStat

## Abbreviations

CPU: Central processing unit
HTML: Hypertext markup language
Mac: Macintosh
NCBI: National Center for Biotechnology Information
NGS: Next generation sequencing
QC: Quality control
SRA: Sequence read archive

## References

[1] Goodwin S, McPherson JD, McCombie WR (2016). Coming of age: ten years of next-generation sequencing technologies. Nat Rev Genet.

[2] Kodama Y, Shumway M, Leinonen R (2012). International nucleotide sequence database C: the sequence read archive: explosive growth of sequencing data. Nucleic Acids Res.

[3] Park ST, Kim J (2016). Trends in next-generation sequencing and a new era for whole genome sequencing. Int Neurourol J.

[4] Tagu D, Colbourne JK, Negre N (2014). Genomic data integration for ecological and evolutionary traits in non-model organisms. BMC Genomics.

[5] da Fonseca RR, Albrechtsen A, Themudo GE, Ramos-Madrigal J, Sibbesen JA, Maretty L, Zepeda-Mendoza ML, Campos PF, Heller R, Pereira RJ (2016). Next-generation biology: sequencing and data analysis approaches for non-model organisms. Mar Genomics.

[6] Gullapalli RR, Desai KV, Santana-Santos L, Kant JA, Becich MJ (2012). Next generation sequencing in clinical medicine: challenges and lessons for pathology and biomedical informatics. J Pathol Inform.

[7] Motro Y, Moran-Gilad J (2017). Next-generation sequencing applications in clinical bacteriology. Biomol Detect Quantif.

[8] Manini TM, Buford TW, Kairalla JA, McDermott MM, Vaz Fragoso CA, Fielding RA, Hsu FC, Johannsen N, Kritchevsky S, Harris TB, et al. Meta-analysis identifies mitochondrial DNA sequence variants associated with walking speed. Geroscience. 2018. 10.1007/s11357-018-0043-x PMC6294723 30338417

[9] Beigh Mohammad (2016). Next-Generation Sequencing: The Translational Medicine Approach from “Bench to Bedside to Population”. Medicines.

[10] Wetterstrand KA (2018). DNA sequencing costs: data from the NHGRI genome sequencing program (GSP). In: *wwwgenomegov/sequencingcostsdata*.

[11] Dahlo M, Scofield DG, Schaal W, Spjuth O. Tracking the NGS revolution: managing life science research on shared high-performance computing clusters. Gigascience. 2018;7(5). 10.1093/gigascience/giy028 PMC5928410 29659792

[12] Muir P, Li S, Lou S, Wang D, Spakowicz DJ, Salichos L, Zhang J, Weinstock GM, Isaacs F, Rozowsky J (2016). The real cost of sequencing: scaling computation to keep pace with data generation. Genome Biol.

[13] Merino GA, Fresno C, Netto F, Netto ED, Pratto L, Fernández EA (2016). The impact of quality control in RNA-seq experiments. J Phys Conf Ser.

[14] Shcherbina A (2014). FASTQSim: platform-independent data characterization and in silico read generation for NGS datasets. BMC Res Notes.

[15] Davis MP, van Dongen S, Abreu-Goodger C, Bartonicek N, Enright AJ (2013). Kraken: a set of tools for quality control and analysis of high-throughput sequence data. Methods.

[16] Kalari KR, Nair AA, Bhavsar JD, O'Brien DR, Davila JI, Bockol MA, Nie J, Tang X, Baheti S, Doughty JB (2014). MAP-RSeq: Mayo analysis pipeline for RNA sequencing. BMC Bioinformatics.

[17] Lavender CA, Shapiro AJ, Burkholder AB, Bennett BD, Adelman K, Fargo DC (2017). ORIO (online resource for integrative omics): a web-based platform for rapid integration of next generation sequencing data. Nucleic Acids Res.

[18] Aevermann B, McCorrison J, Venepally P, Hodge R, Bakken T, Miller J, Novotny M, Tran DN, Diezfuertes F, Christiansen L (2017). Production of a preliminary quality control pipeline for single nuclei Rna-Seq and its application in the analysis of cell type diversity of post-mortem human brain neocortex. Pac Symp Biocomput.

[19] Li Bingshan, Zhan Xiaowei, Wing Mary-Kate, Anderson Paul, Kang Hyun Min, Abecasis Goncalo R. (2013). QPLOT: A Quality Assessment Tool for Next Generation Sequencing Data. BioMed Research International.

[20] Cabanski CR, Cavin K, Bizon C, Wilkerson MD, Parker JS, Wilhelmsen KC, Perou CM, Marron JS, Hayes DN (2012). ReQON: a Bioconductor package for recalibrating quality scores from next-generation sequencing data. BMC Bioinformatics.

[21] Peng Y, Maxwell AS, Barker ND, Laird JG, Kennedy AJ, Wang N, Zhang C, Gong P (2014). SeqAssist: a novel toolkit for preliminary analysis of next-generation sequencing data. BMC Bioinformatics.

[22] Hong C, Manimaran S, Johnson WE (2014). PathoQC: computationally efficient read preprocessing and quality control for high-throughput sequencing data sets. Cancer Inform.

[23] Katta MA, Khan AW, Doddamani D, Thudi M, Varshney RK (2015). NGS-QCbox and raspberry for parallel, automated and rapid quality control analysis of large-scale next generation sequencing (Illumina) data. PLoS One.

[24] Knowles DG, Roder M, Merkel A, Guigo R (2013). Grape RNA-Seq analysis pipeline environment. Bioinformatics.

[25] Lo CC, Chain PS (2014). Rapid evaluation and quality control of next generation sequencing data with FaQCs. BMC Bioinformatics.

[26] Pandey RV, Pabinger S, Kriegner A, Weinhausel A (2016). ClinQC: a tool for quality control and cleaning of sanger and NGS data in clinical research. BMC Bioinformatics.

[27] Patel RK, Jain M (2012). NGS QC toolkit: a toolkit for quality control of next generation sequencing data. PLoS One.

[28] Zhou Q, Su X, Jing G, Chen S, Ning K (2018). RNA-QC-chain: comprehensive and fast quality control for RNA-Seq data. BMC Genomics.

[29] Auer PL, Doerge RW (2010). Statistical design and analysis of RNA sequencing data. Genetics.

[30] Andrews S: FastQC: a quality control tool for high throughput sequence data. Reference Source; 2010. https://www.bioinformatics.babraham.ac.uk/projects/fastqc/.

[31] Bolger AM, Lohse M, Usadel B (2014). Trimmomatic: a flexible trimmer for Illumina sequence data. Bioinformatics.

