# Supplementary material for: FQStat: a parallel architecture for very high-speed assessment of sequencing quality metrics
Source: BMC Bioinformatics. 2019 Aug 15;20:424. doi: 10.1186/s12859-019-3015-y (PMC6694608; doi:10.1186/s12859-019-3015-y)
Supplement: Supplementary file 3 — Effect of file split size and number of cores per file. Read Length = 150 bp (PDF 1462 kb) [file 12859_2019_3015_MOESM3_ESM.pdf]

Supplementary File 3

Effect of file split size and number of cores per file

Read Length = 150 bp

DATA RANGE

File size (number of reads): 10^6, 2×10^6, 3×10^6, 4×10^6, 5×10^6, 10^7, 1.5×10^7, 2×10^7, 3×10^7, 5×10^7, 10^8

Segment size (number of reads): 10^5, 2×10^5, 5×10^5, 10^6, 2×10^6, 5×10^6, 10^7, 3×10^7, 5×10^7, 10^8

Number of cores: 10, 16, 20, 30, 40, 50, 60, 70, 80, 90, 100, 120, 140

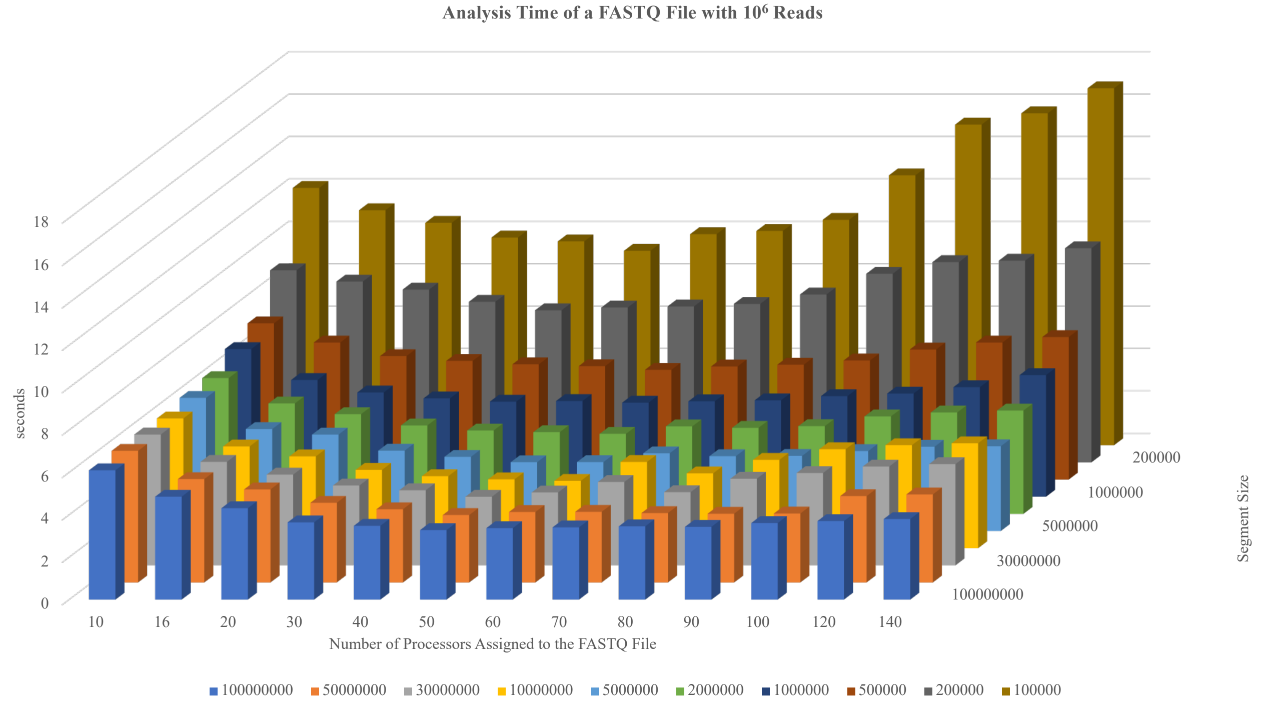

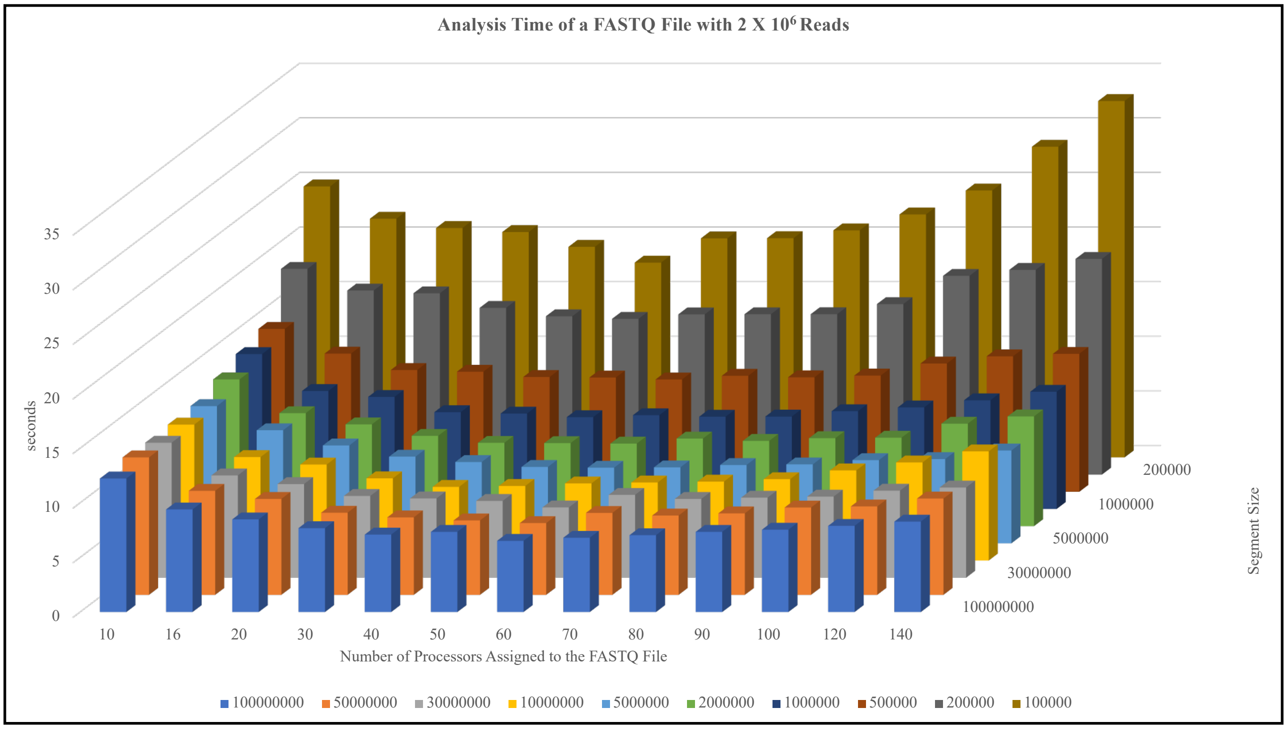
Analysis Time of a FASTQ File with 2 X 10⁶ Reads
seconds
0
5
10
15
20
25
30
35
10
16
20
30
40
50
60
70
80
90
100
120
140
Number of Processors Assigned to the FASTQ File
Segment Size
100000000
30000000
5000000
1000000
200000
100000000
50000000
30000000
10000000
5000000
2000000
1000000
500000
200000
100000

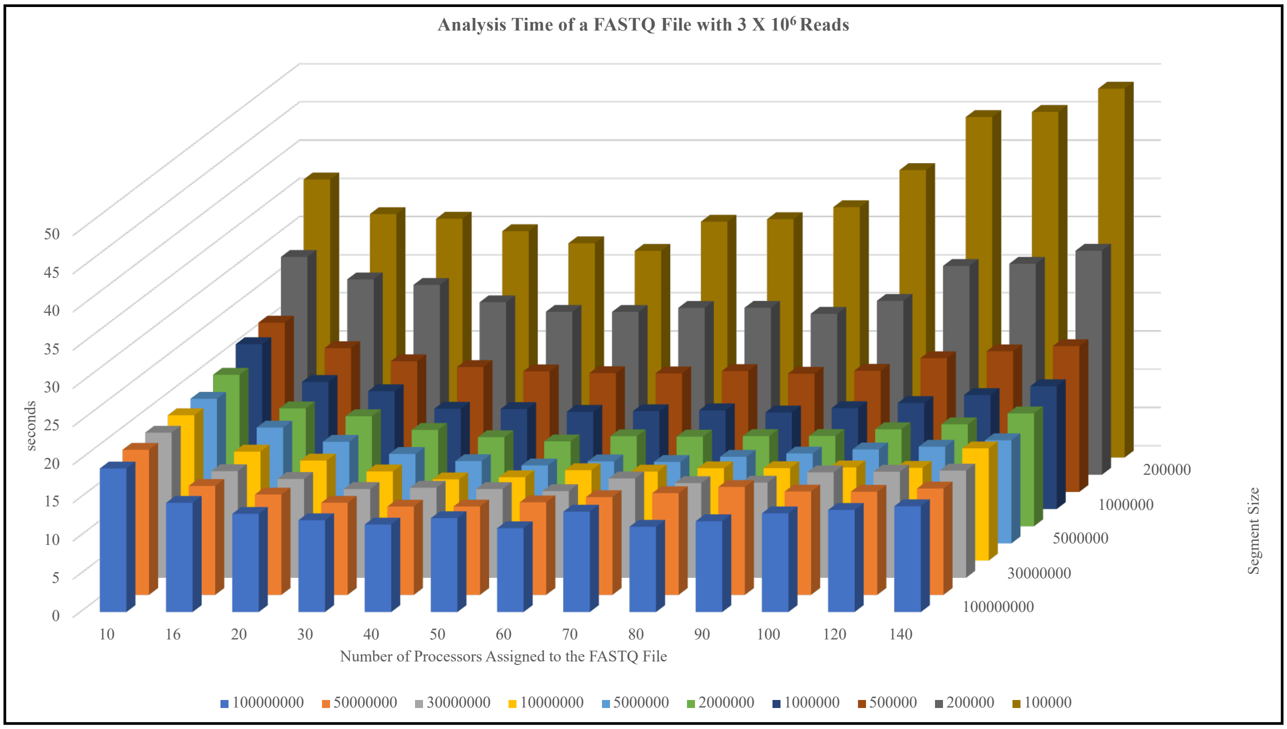
Analysis Time of a FASTQ File with 3 X 10⁶ Reads
seconds
0
5
10
15
20
25
30
35
40
45
50
10
16
20
30
40
50
60
70
80
90
100
120
140
Number of Processors Assigned to the FASTQ File
Segment Size
100000000
30000000
5000000
1000000
200000
100000000
50000000
30000000
10000000
5000000
2000000
1000000
500000
200000
100000

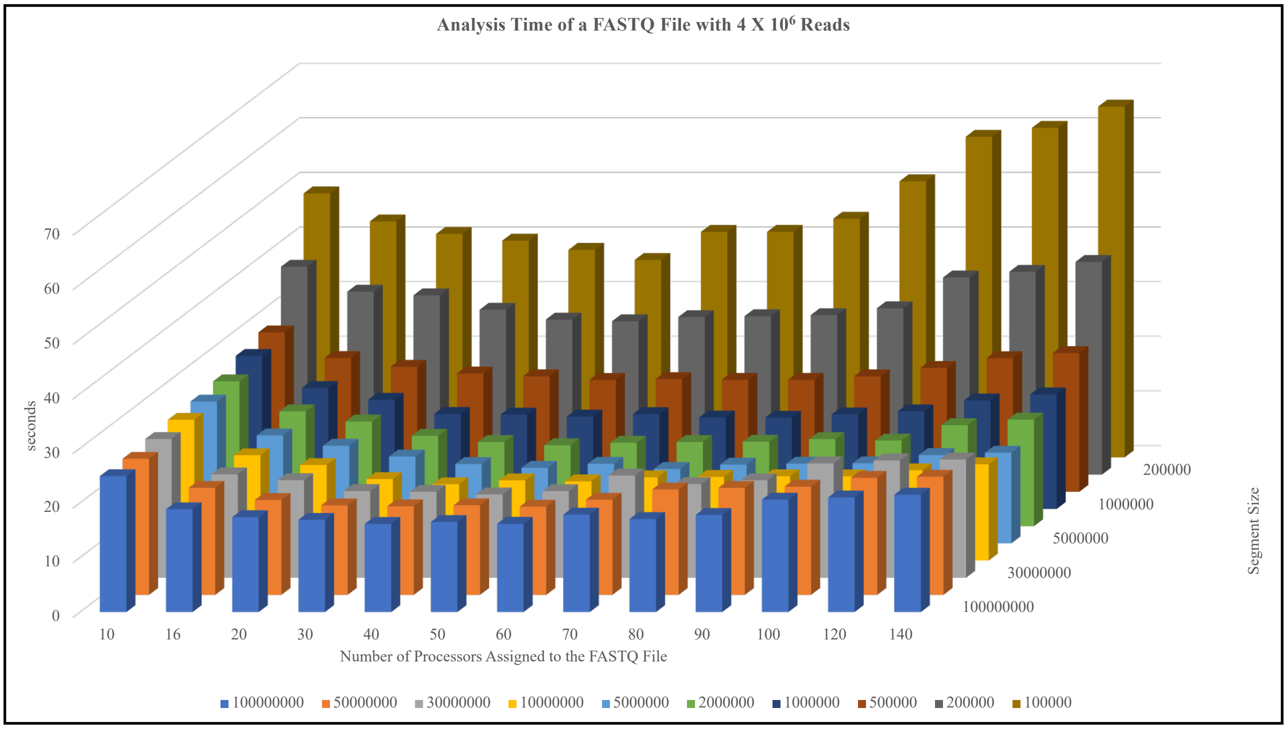
Analysis Time of a FASTQ File with 4 X 10^6 Reads
seconds
0
10
20
30
40
50
60
70
10
16
20
30
40
50
60
70
80
90
100
120
140
Number of Processors Assigned to the FASTQ File
200000
1000000
5000000
30000000
100000000
Segment Size
100000000
50000000
30000000
10000000
5000000
2000000
1000000
500000
200000
100000

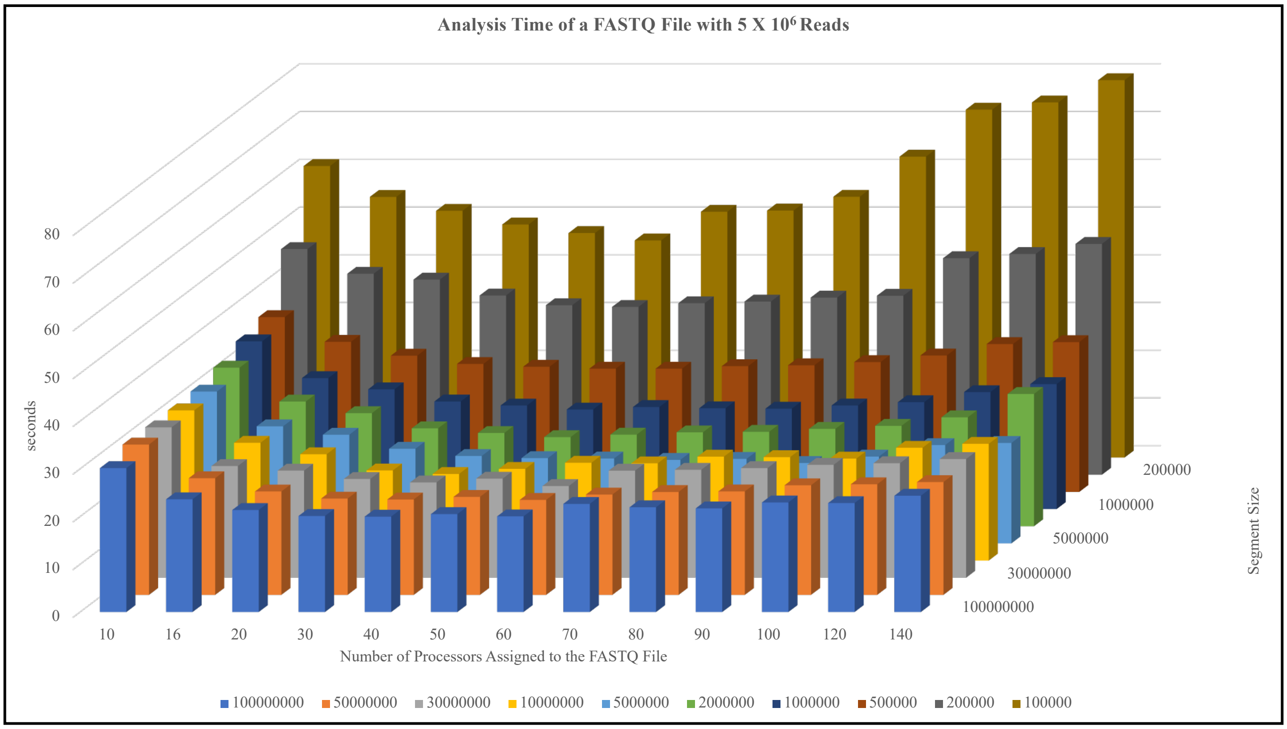
Analysis Time of a FASTQ File with 5 X 10^6 Reads
seconds
0
10
20
30
40
50
60
70
80
10
16
20
30
40
50
60
70
80
90
100
120
140
Number of Processors Assigned to the FASTQ File
200000
1000000
5000000
30000000
100000000
Segment Size
100000000
50000000
30000000
10000000
5000000
2000000
1000000
500000
200000
100000

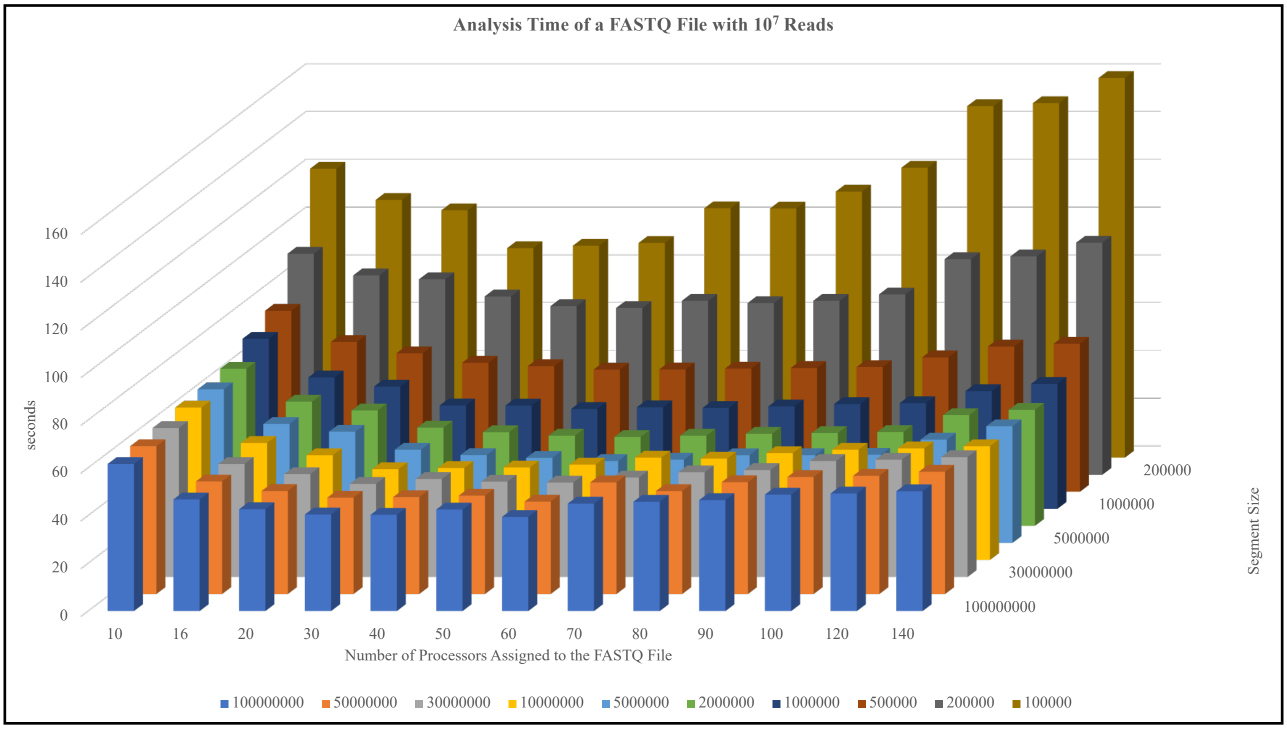
Analysis Time of a FASTQ File with 10^7 Reads
seconds
0
20
40
60
80
100
120
140
160
10
16
20
30
40
50
60
70
80
90
100
120
140
Number of Processors Assigned to the FASTQ File
100000000
30000000
5000000
1000000
200000
Segment Size
100000000
50000000
30000000
10000000
5000000
2000000
1000000
500000
200000
100000

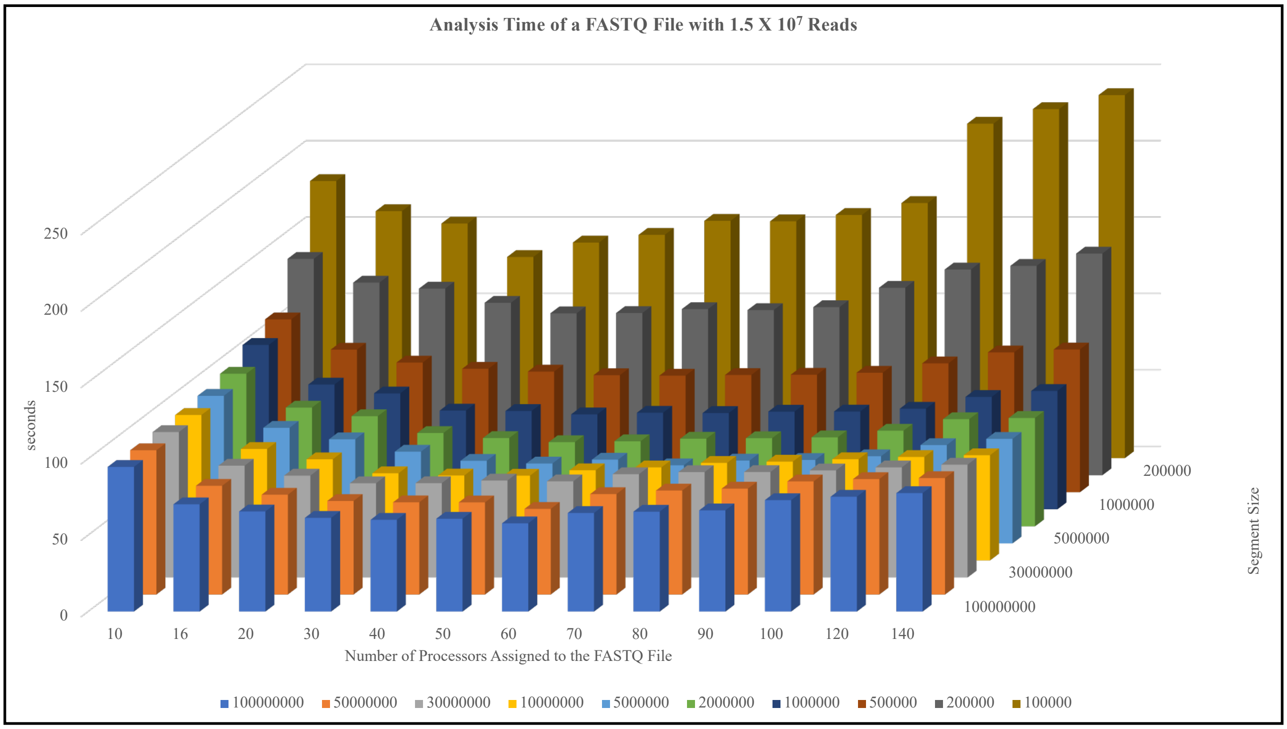
Analysis Time of a FASTQ File with 1.5 X 10^7 Reads
seconds
0
50
100
150
200
250
10
16
20
30
40
50
60
70
80
90
100
120
140
Number of Processors Assigned to the FASTQ File
100000000
30000000
5000000
1000000
200000
Segment Size
100000000
50000000
30000000
10000000
5000000
2000000
1000000
500000
200000
100000

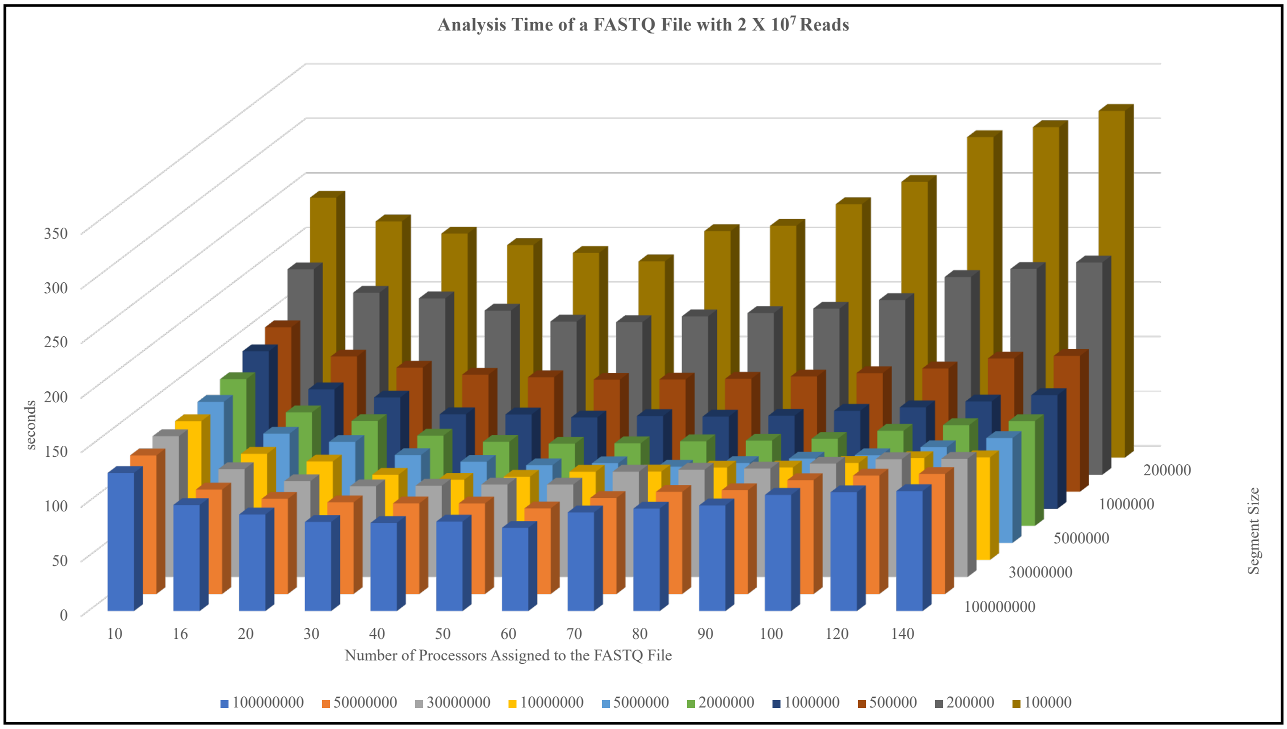
Analysis Time of a FASTQ File with 2 X 10^7 Reads
seconds
0
50
100
150
200
250
300
350
10
16
20
30
40
50
60
70
80
90
100
120
140
Number of Processors Assigned to the FASTQ File
Segment Size
100000000
30000000
5000000
1000000
200000
100000000
50000000
30000000
10000000
5000000
2000000
1000000
500000
200000
100000

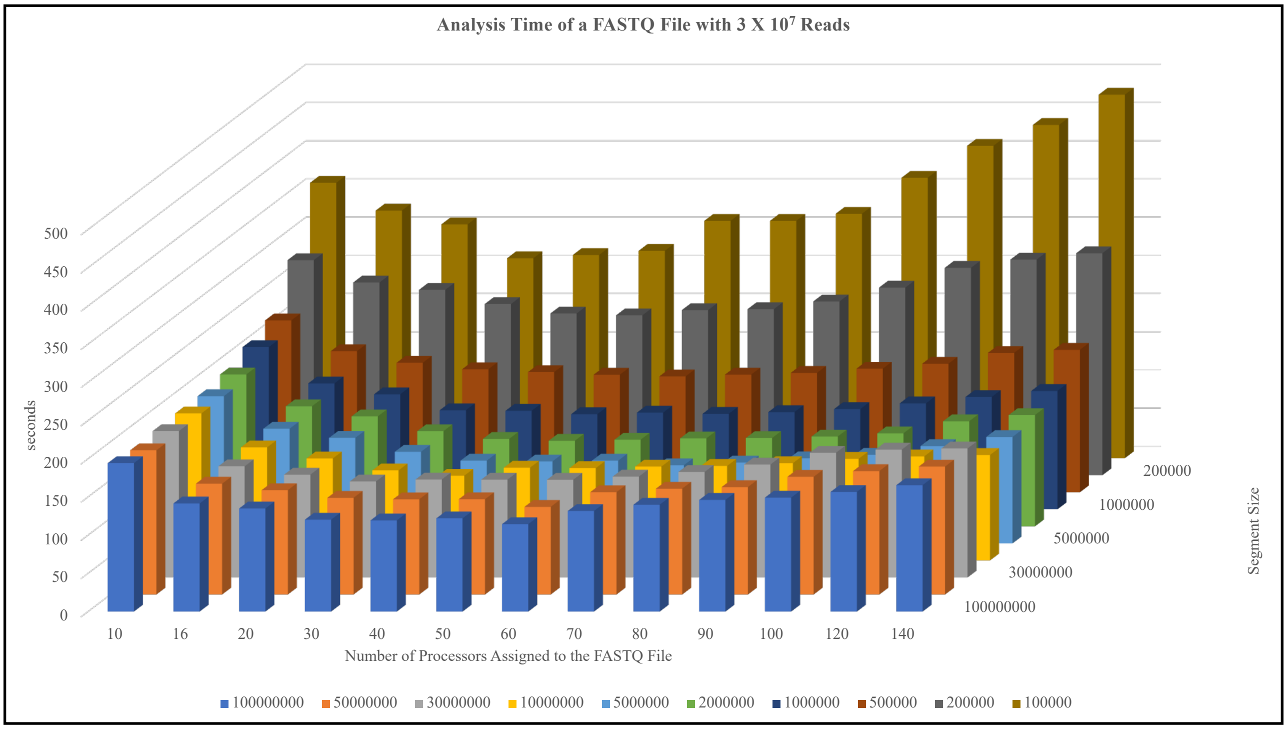
Analysis Time of a FASTQ File with 3 X 10^7 Reads
seconds
0
50
100
150
200
250
300
350
400
450
500
10
16
20
30
40
50
60
70
80
90
100
120
140
Number of Processors Assigned to the FASTQ File
Segment Size
100000000
30000000
5000000
1000000
200000
100000000
50000000
30000000
10000000
5000000
2000000
1000000
500000
200000
100000

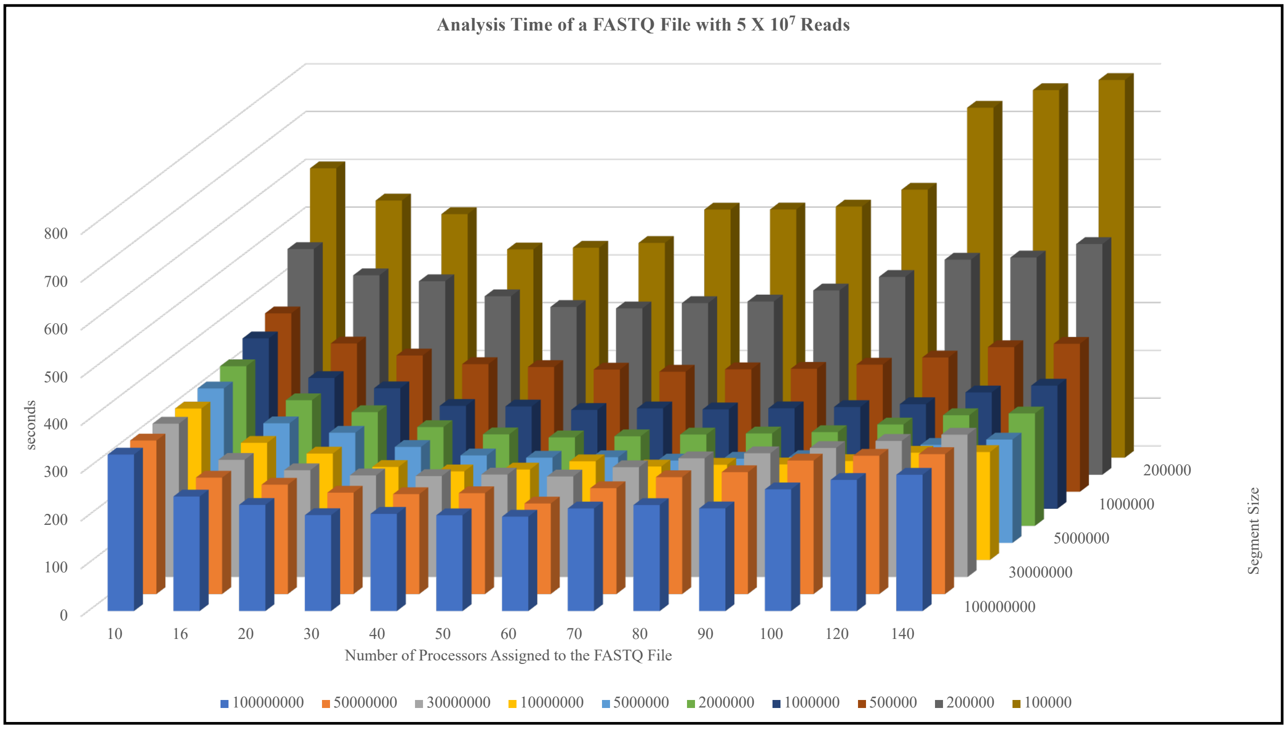

Analysis Time of a FASTQ File with 5 X 10^7 Reads
seconds
0
100
200
300
400
500
600
700
800
10 16 20 30 40 50 60 70 80 90 100 120 140
Number of Processors Assigned to the FASTQ File
Segment Size
100000000
30000000
5000000
1000000
200000
100000000 50000000 30000000 10000000 5000000 2000000 1000000 500000 200000 100000

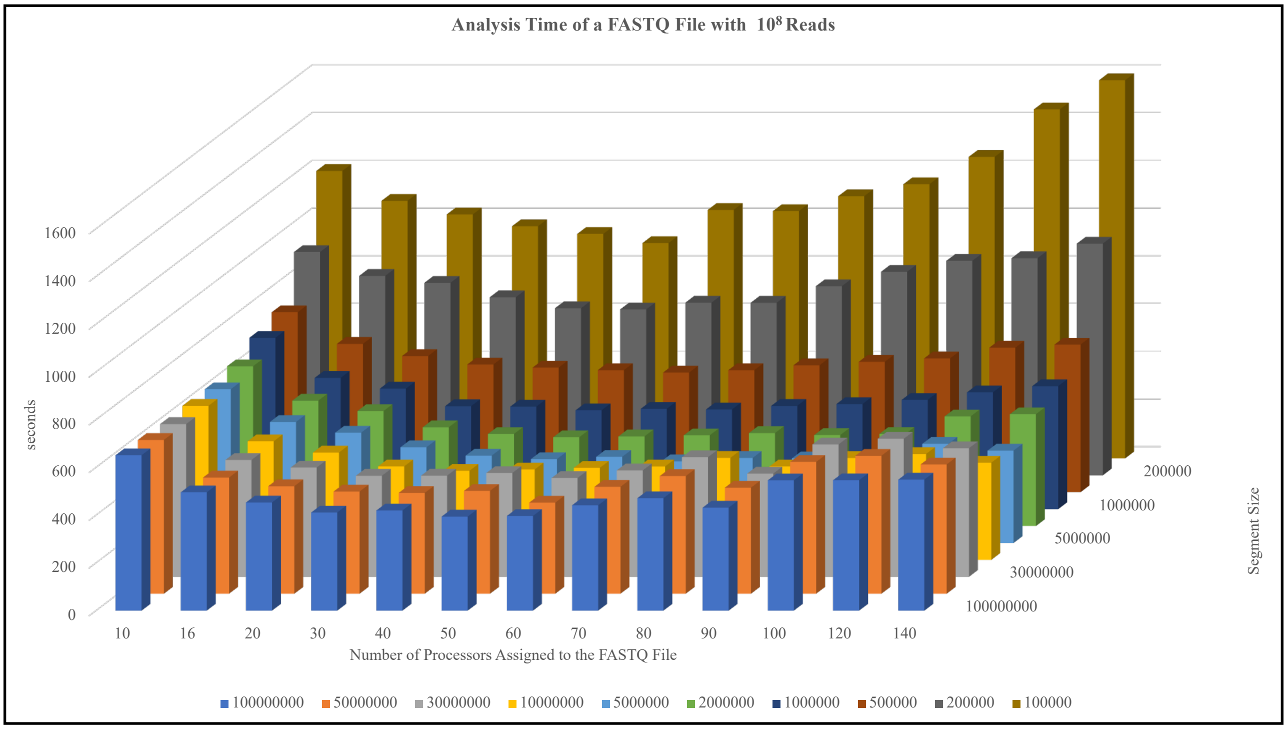

Analysis Time of a FASTQ File with 10^8 Reads
seconds
0
200
400
600
800
1000
1200
1400
1600
10 16 20 30 40 50 60 70 80 90 100 120 140
Number of Processors Assigned to the FASTQ File
Segment Size
100000000
30000000
5000000
1000000
200000
100000000 50000000 30000000 10000000 5000000 2000000 1000000 500000 200000 100000
