# Supplementary material for: FQStat: a parallel architecture for very high-speed assessment of sequencing quality metrics
Source: BMC Bioinformatics. 2019 Aug 15;20:424. doi: 10.1186/s12859-019-3015-y (PMC6694608; doi:10.1186/s12859-019-3015-y)
Supplement: Supplementary file 4 — Performance gain per assigned core. Read Length = 75 bp (PDF 1079 kb) [file 12859_2019_3015_MOESM4_ESM.pdf]

Supplementary File 4

Performance gain per assigned core

Read Length = 75 bp

DATA RANGE

File size (number of reads): 10^6, 2 x 10^6, 3 x 10^6, 4 x 10^6, 5 x 10^6, 10^7, 1.5 x 10^7, 2 x 10^7, 3 x 10^7, 5 x 10^7, 10^8

Segment size (number of reads): 10^5, 2 x 10^5, 5 x 10^5, 10^6, 2 x 10^6, 5 x 10^6, 10^7, 3 x 10^7, 5 x 10^7, 10^8

Number of cores: 10, 16, 20, 30, 40, 50, 60, 70, 80, 90, 100, 120, 140

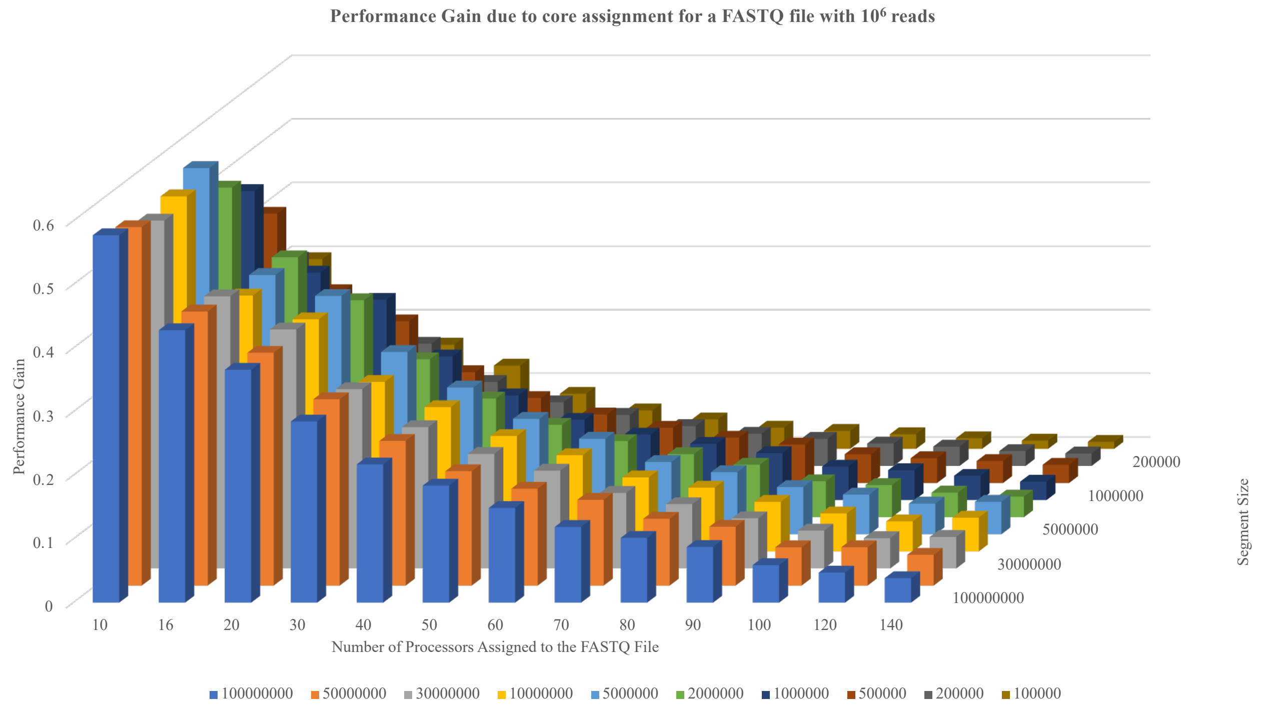

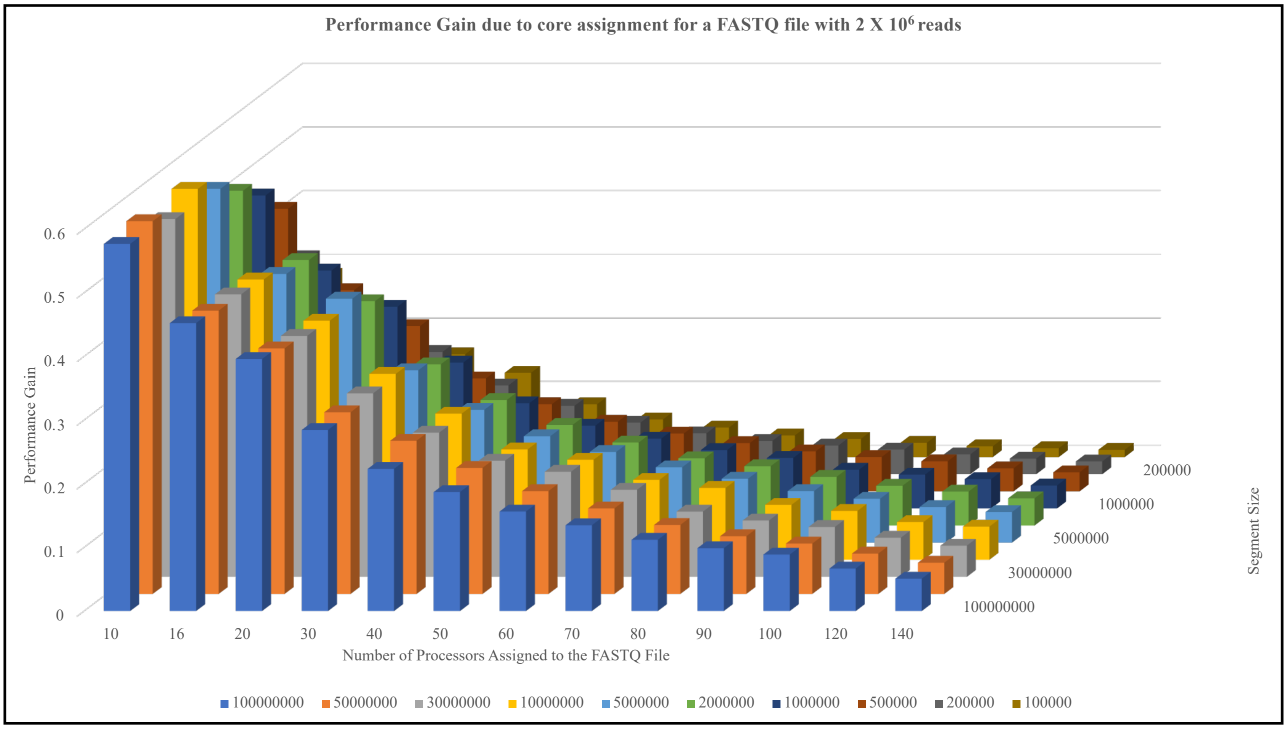
Performance Gain due to core assignment for a FASTQ file with 2 X 10^6 reads
Performance Gain
0
0.1
0.2
0.3
0.4
0.5
0.6
10
16
20
30
40
50
60
70
80
90
100
120
140
Number of Processors Assigned to the FASTQ File
100000000
30000000
5000000
1000000
200000
Segment Size
100000000
50000000
30000000
10000000
5000000
2000000
1000000
500000
200000
100000

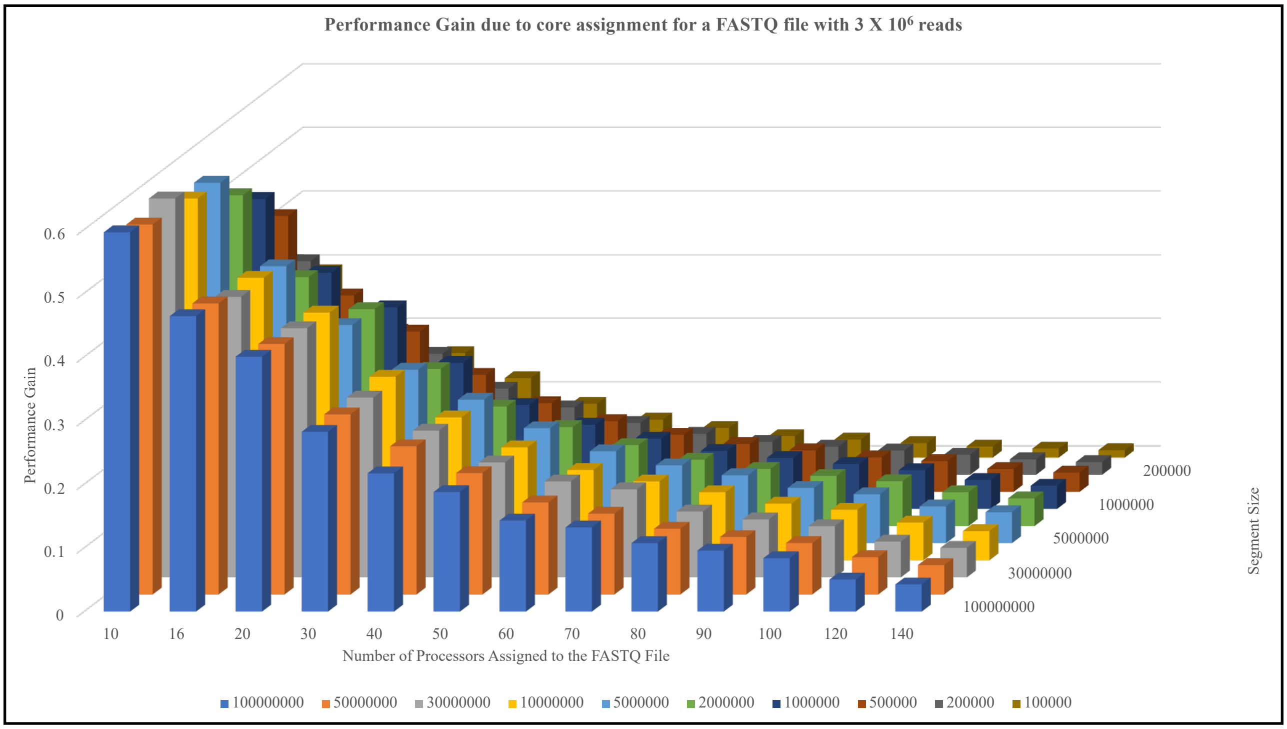
Performance Gain due to core assignment for a FASTQ file with 3 X 10^6 reads
Performance Gain
0
0.1
0.2
0.3
0.4
0.5
0.6
10
16
20
30
40
50
60
70
80
90
100
120
140
Number of Processors Assigned to the FASTQ File
100000000
30000000
5000000
1000000
200000
Segment Size
100000000
50000000
30000000
10000000
5000000
2000000
1000000
500000
200000
100000

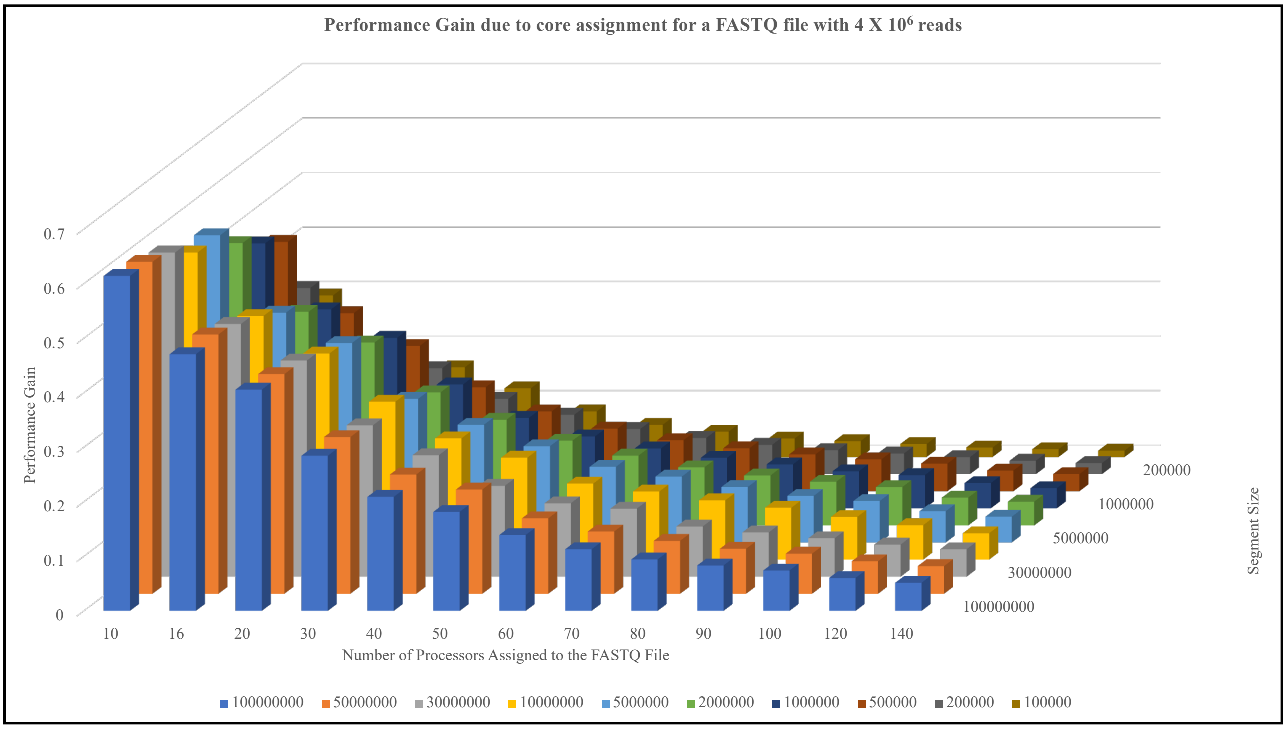

Performance Gain due to core assignment for a FASTQ file with 4 X 10⁶ reads
Performance Gain
0.7
0.6
0.5
0.4
0.3
0.2
0.1
0
10
16
20
30
40
50
60
70
80
90
100
120
140
Number of Processors Assigned to the FASTQ File
200000
1000000
5000000
30000000
100000000
Segment Size
100000000
50000000
30000000
10000000
5000000
2000000
1000000
500000
200000
100000

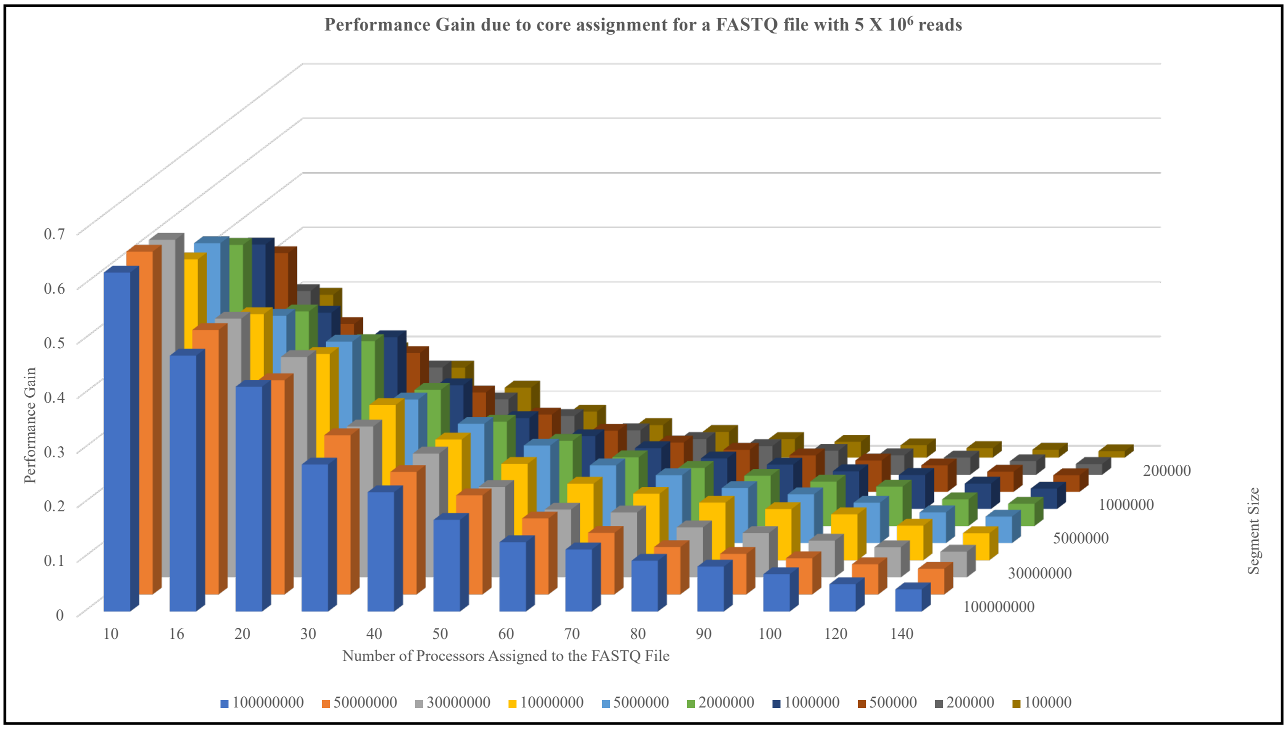

Performance Gain due to core assignment for a FASTQ file with 5 X 10⁶ reads
Performance Gain
0.7
0.6
0.5
0.4
0.3
0.2
0.1
0
10
16
20
30
40
50
60
70
80
90
100
120
140
Number of Processors Assigned to the FASTQ File
200000
1000000
5000000
30000000
100000000
Segment Size
100000000
50000000
30000000
10000000
5000000
2000000
1000000
500000
200000
100000

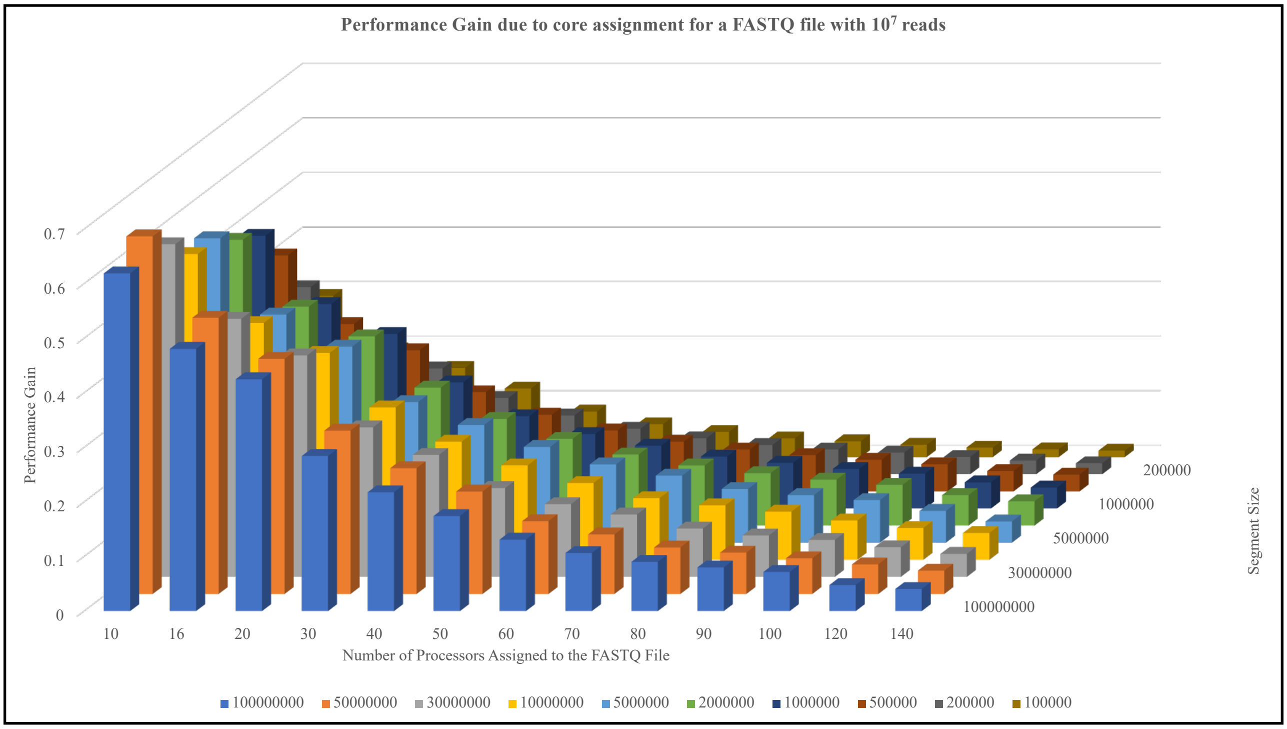

Performance Gain due to core assignment for a FASTQ file with 10^7 reads
Performance Gain
0.7
0.6
0.5
0.4
0.3
0.2
0.1
0
10 16 20 30 40 50 60 70 80 90 100 120 140
Number of Processors Assigned to the FASTQ File
Segment Size
200000
1000000
5000000
30000000
100000000
100000000 50000000 30000000 10000000 5000000 2000000 1000000 500000 200000 100000

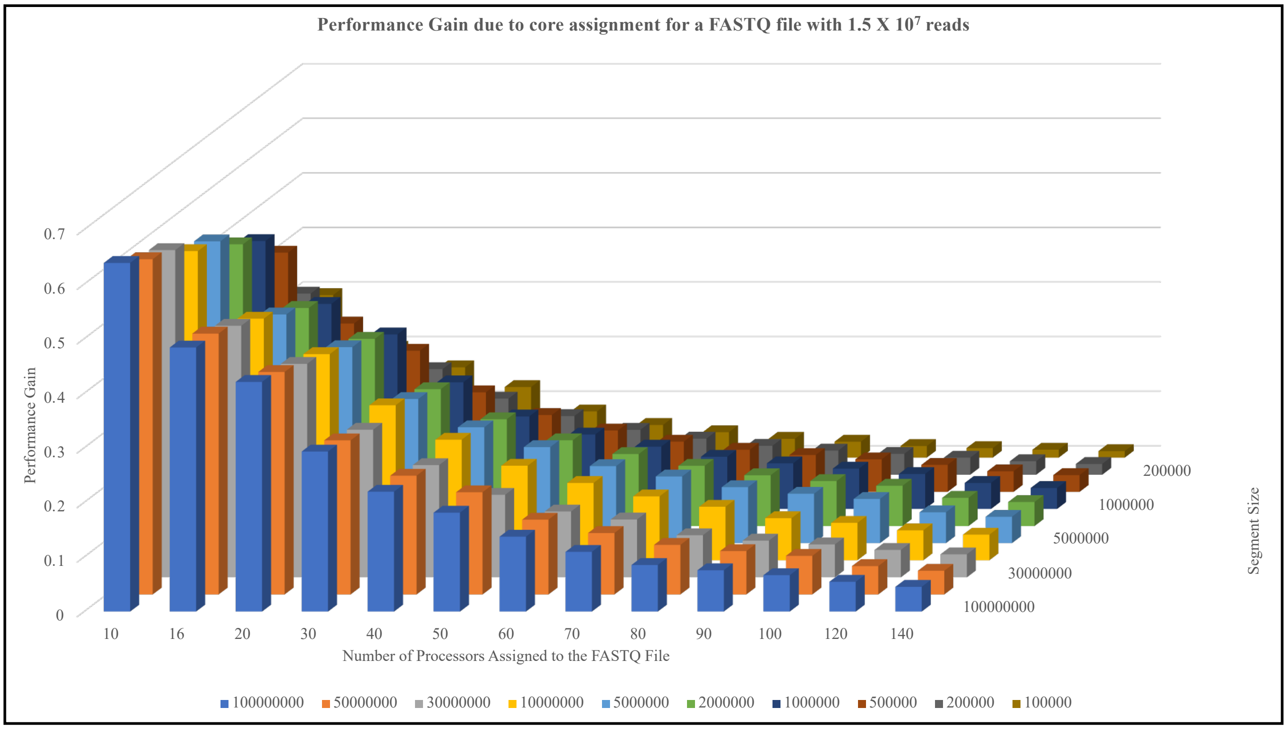

Performance Gain due to core assignment for a FASTQ file with 1.5 X 10^7 reads
Performance Gain
0.7
0.6
0.5
0.4
0.3
0.2
0.1
0
10 16 20 30 40 50 60 70 80 90 100 120 140
Number of Processors Assigned to the FASTQ File
Segment Size
200000
1000000
5000000
30000000
100000000
100000000 50000000 30000000 10000000 5000000 2000000 1000000 500000 200000 100000

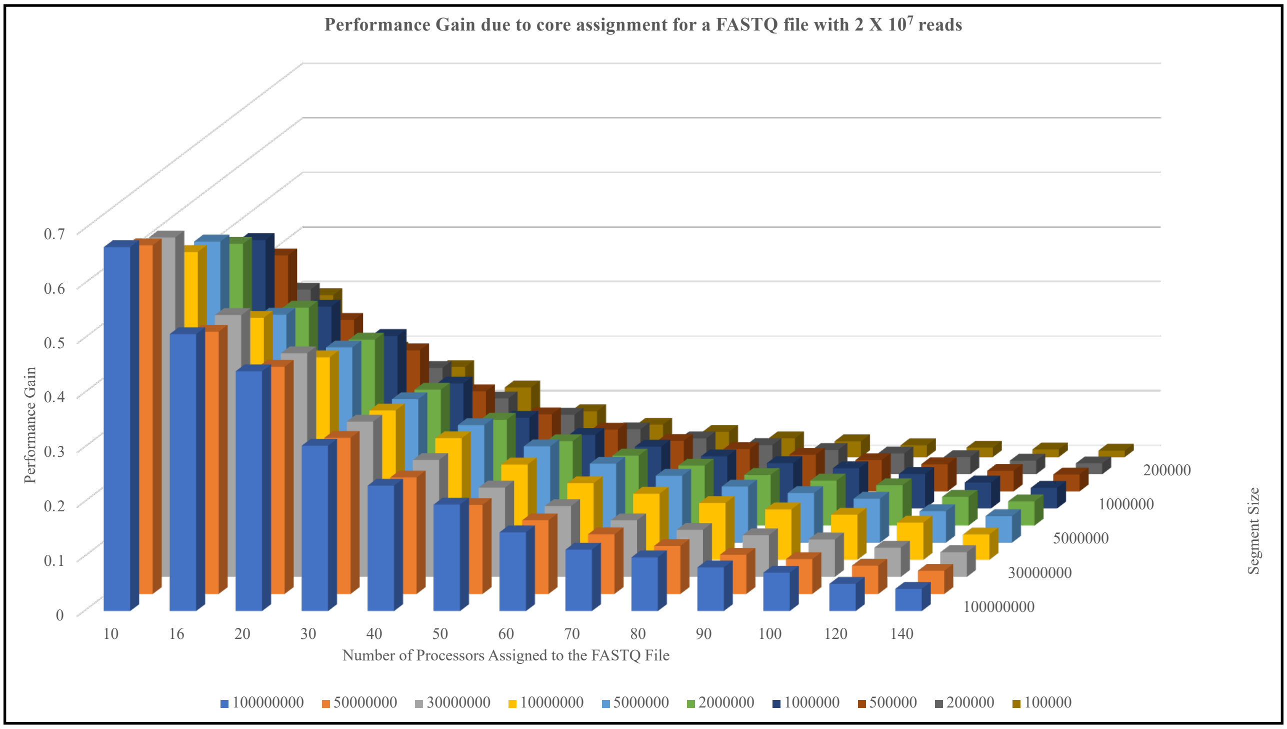
Performance Gain due to core assignment for a FASTQ file with 2 X 10⁷ reads
Performance Gain
0
0.1
0.2
0.3
0.4
0.5
0.6
0.7
10
16
20
30
40
50
60
70
80
90
100
120
140
Number of Processors Assigned to the FASTQ File
Segment Size
100000000
30000000
5000000
1000000
200000
100000000
50000000
30000000
10000000
5000000
2000000
1000000
500000
200000
100000

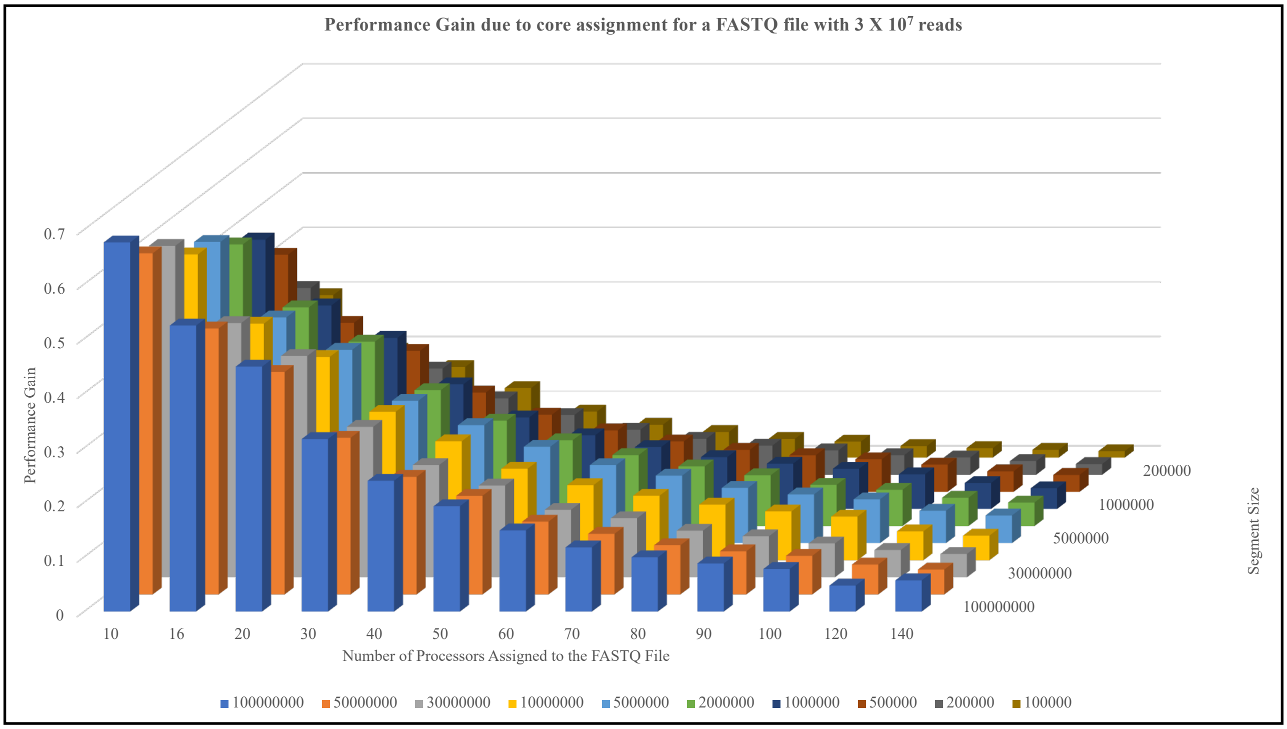
Performance Gain due to core assignment for a FASTQ file with 3 X 10⁷ reads
Performance Gain
0
0.1
0.2
0.3
0.4
0.5
0.6
0.7
10
16
20
30
40
50
60
70
80
90
100
120
140
Number of Processors Assigned to the FASTQ File
Segment Size
100000000
30000000
5000000
1000000
200000
100000000
50000000
30000000
10000000
5000000
2000000
1000000
500000
200000
100000

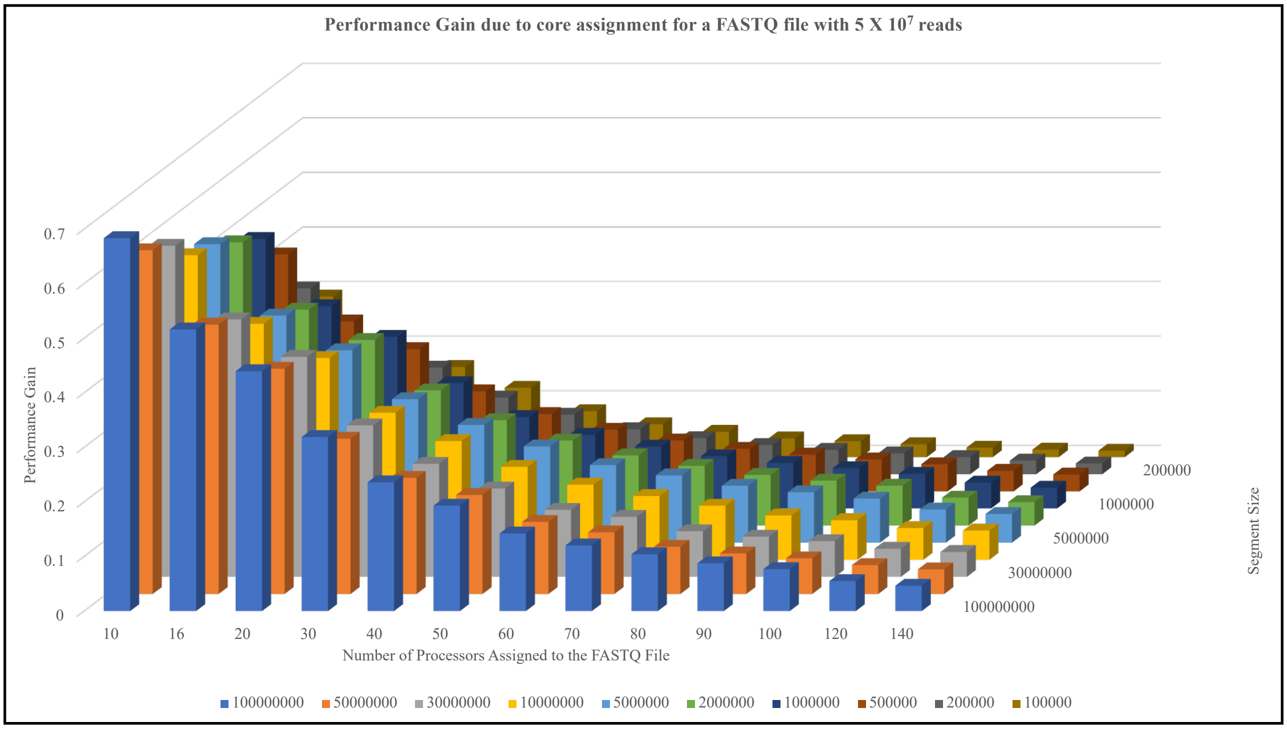

Performance Gain due to core assignment for a FASTQ file with 5 X 10^7 reads
Performance Gain
0
0.1
0.2
0.3
0.4
0.5
0.6
0.7
10
16
20
30
40
50
60
70
80
90
100
120
140
Number of Processors Assigned to the FASTQ File
Segment Size
100000000
30000000
5000000
1000000
200000
100000000
50000000
30000000
10000000
5000000
2000000
1000000
500000
200000
100000

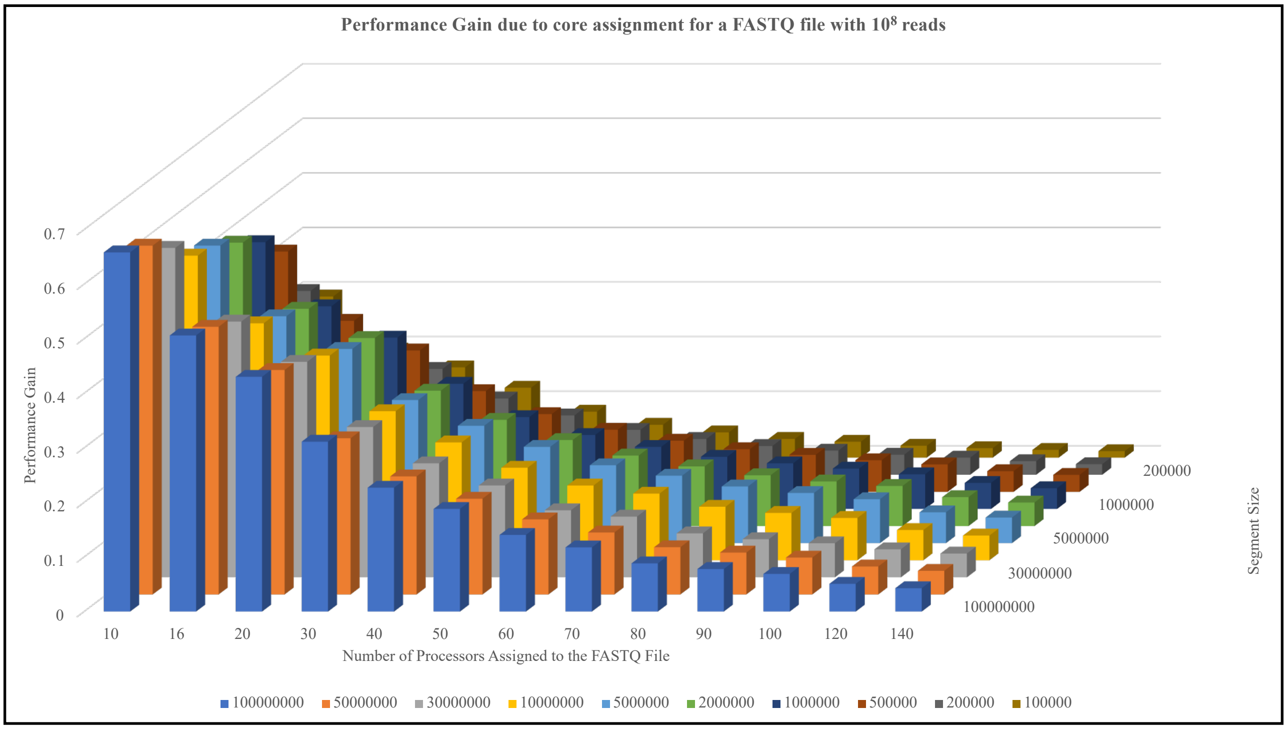

Performance Gain due to core assignment for a FASTQ file with 10^8 reads
Performance Gain
0
0.1
0.2
0.3
0.4
0.5
0.6
0.7
10
16
20
30
40
50
60
70
80
90
100
120
140
Number of Processors Assigned to the FASTQ File
Segment Size
100000000
30000000
5000000
1000000
200000
100000000
50000000
30000000
10000000
5000000
2000000
1000000
500000
200000
100000
